# The ClpX chaperone controls autolytic splitting of *Staphylococcus aureus* daughter cells, but is bypassed by β-lactam antibiotics or inhibitors of WTA biosynthesis

**DOI:** 10.1371/journal.ppat.1008044

**Published:** 2019-09-13

**Authors:** Camilla Jensen, Kristoffer T. Bæk, Clement Gallay, Ida Thalsø-Madsen, Lijuan Xu, Ambre Jousselin, Fernando Ruiz Torrubia, Wilhelm Paulander, Ana R. Pereira, Jan-Willem Veening, Mariana G. Pinho, Dorte Frees

**Affiliations:** 1 Department of Veterinary and Animal Sciences, University of Copenhagen, Copenhagen, Denmark; 2 Department of Fundamental Microbiology, Faculty of Biology and Medicine, University of Lausanne, Lausanne, Switzerland; 3 Bacterial Cell Biology, Instituto de Tecnologia Química e Biológica António Xavier, Universidade Nova de Lisboa, Oeiras, Portugal; National Institutes of Health, UNITED STATES

## Abstract

β-lactam antibiotics interfere with cross-linking of the bacterial cell wall, but the killing mechanism of this important class of antibiotics is not fully understood. Serendipitously we found that sub-lethal doses of β-lactams rescue growth and prevent spontaneous lysis of *Staphylococcus aureus* mutants lacking the widely conserved chaperone ClpX, and we reasoned that a better understanding of the *clpX* phenotypes could provide novel insights into the downstream effects of β-lactam binding to the PBP targets. Super-resolution imaging revealed that *clpX* cells display aberrant septum synthesis, and initiate daughter cell separation prior to septum completion at 30°C, but not at 37°C, demonstrating that ClpX becomes critical for coordinating the *S*. *aureus* cell cycle as the temperature decreases. FtsZ localization and dynamics were not affected in the absence of ClpX, suggesting that ClpX affects septum formation and autolytic activation downstream of Z-ring formation. Interestingly, oxacillin antagonized the septum progression defects of *clpX* cells and prevented lysis of prematurely splitting *clpX* cells. Strikingly, inhibitors of wall teichoic acid (WTA) biosynthesis that work synergistically with β-lactams to kill MRSA synthesis also rescued growth of the *clpX* mutant, as did genetic inactivation of the gene encoding the septal autolysin, Sle1. Taken together, our data support a model in which Sle1 causes premature splitting and lysis of *clpX* daughter cells unless Sle1-dependent lysis is antagonized by β-lactams or by inhibiting an early step in WTA biosynthesis. The finding that β-lactams and inhibitors of WTA biosynthesis specifically prevent lysis of a mutant with dysregulated autolytic activity lends support to the idea that PBPs and WTA biosynthesis play an important role in coordinating cell division with autolytic splitting of daughter cells, and that β-lactams do not kill *S*. *aureus* simply by weakening the cell wall.

## Author summary

The bacterium *Staphylococcus aureus* is a major cause of human disease, and the rapid spread of *S*. *aureus* strains that are resistant to almost all β-lactam antibiotics has made treatment increasingly difficult. β-lactams interfere with cross-linking of the bacterial cell wall but the killing mechanism of this important class of antibiotics is not fully understood. Here we provide novel insight into this topic by examining a defined *S*. *aureus* mutant that has the unusual property of growing markedly better in the presence of β-lactams. Without β-lactams this mutant dies spontaneously at a high frequency due to premature separation of daughter cells during cell division. Cell death of the mutant can, however, be prevented either by exposure to β-lactam antibiotics or by inhibiting synthesis of wall teichoic acid, a major component of the cell wall in Gram-positive bacteria with a conserved role in activation of autolytic splitting of daughter cells. The finding that β-lactam antibiotics can prevent lysis of a mutant with deregulated activity of autolytic enzymes involved in daughter cell splitting, emphasizes the idea that β-lactams interfere with the coordination between cell division and daughter cell splitting, and do not kill *S*. *aureus* simply by weakening the cell wall.

## Introduction

*Staphylococcus aureus* is a commensal bacterium capable of causing a variety of both localized and invasive infections. Due to its ability to acquire resistance to all relevant antibiotics *S*. *aureus* remains a major clinical challenge worldwide [1]. The most challenging antimicrobial resistance issue in *S*. *aureus* has been the dissemination of methicillin-resistant *S*. *aureus* (MRSA) strains that are resistant to almost all β-lactam antibiotics, one of the safest and most widely used classes of antibiotics ever developed [2]. Early work on the mechanism of action of β-lactams culminated in the discovery that penicillin inhibits crosslinking of peptidoglycan (PG), the central component of bacterial cell walls [3]. The enzymes mediating cross-linking of peptidoglycan strands, the targets of penicillin, were therefore designated penicillin binding proteins (PBPs). The realization that penicillin inhibits PG crosslinking led to the classical model in which penicillin-mediated cell lysis is believed to occur as a consequence of a mechanically weakened cell wall incapable of withstanding high intracellular turgor [3,4]. The killing effect of β-lactam antibiotics, however, has turned out to be more complex [5–9], and may even vary between bacteria, as the organization of PG synthesis and the number of PBPs differ widely between bacterial species [10]. Spherical bacteria such as *S*. *aureus* have only one cell wall synthesis machine, and *S*. *aureus* encodes only four PBPs [11]. Notably, MRSA and other Staphylococci have obtained resistance to β-lactams by horizontal acquisition of the *mecA* gene encoding an alternative PBP (PBP2a) that is resistant to inhibition by most β-lactams [12,13]. PBP2a mediated resistance additionally depends on several intrinsic factors that can be targeted by specific compounds to re-sensitize MRSA to β-lactams [14–16]. As an example, inhibitors of wall teichoic acid (WTA) biosynthesis, work synergistically with β-lactams to kill MRSA both *in vitro* and in *in vivo* models of infection, thereby opening a novel paradigm for combination treatment of MRSA [16]. Indeed, a combination strategy pairing β-lactamase inhibitors with β-lactams has proven highly successful in restoring β-lactam efficacy against Gram-negative bacteria [17].

In all living cells molecular chaperones are essential for facilitating unfolding and interactions of proteins. The ClpX chaperone is a highly conserved ATP-dependent chaperone that, additionally to functioning as a classical chaperone, can associate with ClpP to form the ClpXP proteolytic complex [18]. *S*. *aureus clpX* mutant exhibits a mild growth defect at 37°C that is severely exacerbated at 30°C [19,20]. This cold-sensitive growth defect of the *clpX* mutant is independent of ClpP, and is alleviated by loss-of-function mutations in the *ltaS* gene [20,21]. *ltaS* encodes the LtaS synthetase that is required for synthesis of lipoteichoic acid (LTA), an essential cell wall polymer of Gram-positive bacteria controlling cell division and autolytic activity [22]. Interestingly, inactivation of ClpX restored the septum placement defects of cells depleted for LTA, suggesting a link between ClpX and cell division in *S*. *aureus* [20].

Here we demonstrate that ClpX becomes critical for progression of *S*. *aureus* septum synthesis as the temperature decreases. In cells with stalling of septum synthesis, autolytic splitting of daughter cells is activated prior to septum completion resulting in cell lysis, unless β-lactam antibiotics are added to the growth medium. Strikingly, inhibitors of the first step in WTA biosynthesis, similarly to inactivation of the Sle1 autolysin specifically rescues growth of *S*. *aureus clpX* mutants, supporting a fundamental connection between the transpeptidase activity of PBPs, teichoic acids biosynthesis and activation of autolysins mediating septal splitting. In conclusion, this study identifies the ClpX chaperone as an important player in *S*. *aureus* cell division, and provides novel insight into the link between β-lactam antibiotics and cell division in this important pathogen.

## Results

### β-lactam antibiotics stimulate growth of *S*. *aureus clpX* mutants

**Fig 1:**
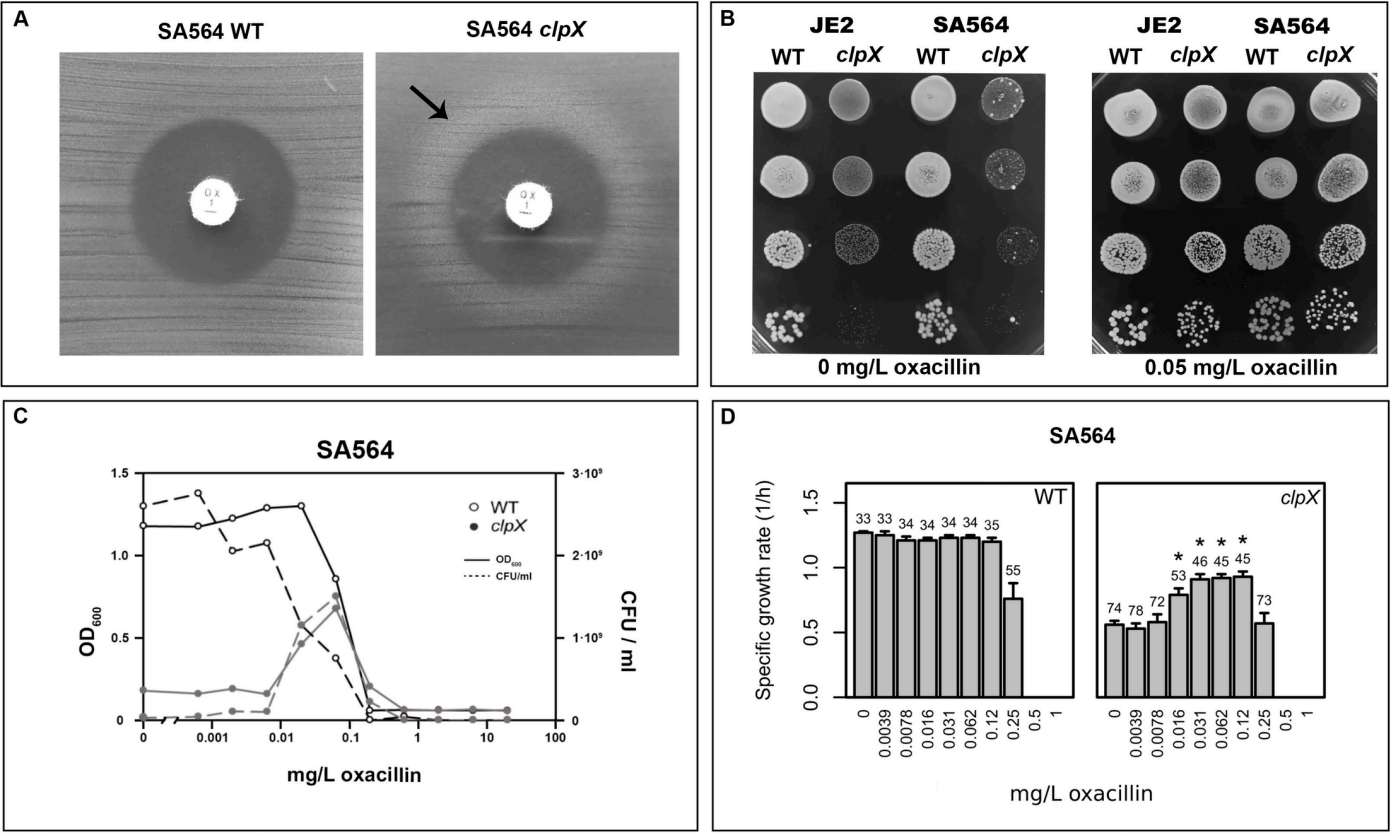
Growth of *S*. *aureus clpX* mutants is stimulated by β-lactams. (**A**) SA564 wild-type and SA564*clpX* were plated at 37°C and tested for susceptibility to oxacillin in a disc diffusion assay; arrow points to zone of improved growth. Disks contain 1 μg oxacillin. (**B**) The *S*. *aureus* wild-type strains, SA564 (MSSA) and JE2 (MRSA) and the corresponding *clpX* deletion mutants were grown exponentially in TSB at 37°C. At OD_600_ = 0.5, the cultures were diluted 10^1^, 10^2^, 10^3^ and 10^4^-fold, and 10 μl of each dilution was spotted on TSA plates +/- oxacillin and the plates were subsequently incubated at 30°C for 24 h. Note that fast growing suppressor colonies appear with high frequency when the SA564 *clpX* cells are plated at 30°C as described in [20]. (**C**) Final yield (OD_600_ or cfu ml^-1^) reached by SA564 wild type and SA564*clpX* cells when grown in microtiter plates for 24 h at 30°C in the absence or presence of increasing concentrations of oxacillin as indicated. (**D**) Growth rates (h^-1^) for *S*. *aureus* SA564 wild-type and SA564*clpX* when grown at 30°C in the absence or presence of increasing concentrations of oxacillin were determined in a Bioscreen C instrument. The average growth rate (h^-1^) and standard deviations from three biological replicates were plotted. Numbers above bars indicate average doubling time in minutes. Asterisks indicate significantly improved growth rate (P < 0.05). The P values were obtained by comparing the growth rates at each concentration to the growth rate without antibiotics and were calculated using Student’s t-test.

Serendipitously, while determining the susceptibility of the *S*. *aureus clpX* mutant to oxacillin, we repeatedly observed zones of improved growth at a certain distance from the filter discs containing the antibiotic, a phenomenon that was not observed for wild-type strains (marked by arrow in Fig 1A). This observation indicated that sub-lethal concentrations of oxacillin stimulate growth of the *clpX* mutant. Indeed, addition of sub-lethal concentrations of oxacillin rescued the severe growth defect normally seen for *S*. *aureus clpX* mutants at 30°C (Fig 1B). To investigate if growth of *S*. *aureus clpX* mutants is generally improved by addition of β-lactam antibiotics, three *S*. *aureus* strains of clinical origin, representing both MRSA (JE2) and methicillin-sensitive *S*. *aureus* (SA564 and Newman), and the corresponding *clpX* deletion strains were grown in broth containing oxacillin, meropenem or cefuroxime (representing three different chemical classes of β-lactams) in various concentrations below and above the previously determined MIC values [23]. We found that the presence of β-lactam antibiotics increased the final yield and growth rate of the *clpX* mutants in all strain backgrounds (Figs 1C and 1D and S1). As shown previously [23], inactivation of *clpX* increased the MIC values in the JE2 background (S1 Fig), but not in the MSSA strain backgrounds. A wide range of β-lactam concentrations was tested, but we did not identify any concentration at which the growth rates of the wild-type strains were enhanced (S1 Fig). For comparison, we also included *clpP* mutants, but observed no or only a minor stimulatory effect on the growth rate of the *clpP* mutants in the presence of β-lactams (S1 Fig). We conclude that the ClpX dependent growth defect that is suppressed by β-lactams is caused by loss of ClpX chaperone activity, not loss of ClpXP protease activity. Given the unusual ability of β-lactams to stimulate growth of the *clpX* mutant, we reasoned that a better understanding of the *clpX* growth defect could provide novel insights into how β-lactam antibiotics interfere with growth of *S*. *aureus*.

### Oxacillin prevents premature growth arrest and spontaneous lysis of *clpX* cells

We first speculated that β-lactam antibiotics may rescue growth of the *clpX* mutant by restoring possible abnormalities in the PG composition of the cell wall. To test this hypothesis, peptidoglycan was purified from the cell wall of wild-type and the *clpX* cells grown at 30°C in the presence or absence of oxacillin, and muropeptides were separated and identified by HPLC. The muropeptide profiles of the wild-type strain and the *clpX* strain, however, turned out to be almost identical, with oxacillin inducing similar changes in the PG structure in both strains (S2 Fig). We next assessed if PBPs amounts or binding activity was changed in cells lacking ClpX. To this end, Bocillin-FL labeling was used to detect PBPs in membrane fractions from SA564 wild-type and *clpX* cells grown at 37°C or 30°C (+/- oxacillin). As the PBP4 signal was very weak in the PBP-profiles, the PBP4 levels were additionally determined by Western blot analysis. Interestingly, while no variation in PBP levels was detected between the SA564 wild-type and *clpX* cells grown at 37°C, the level of PBP2 appeared slightly increased in *clpX* cells as compared to wild-type cells when strains were grown at 30°C (S2 Fig). The presence of sub-MIC oxacillin did, however, not impact PBP-levels. Therefore, β-lactams neither seem to rescue growth of *clpX* cells by correcting an abnormal level of cell wall cross linking, nor by adjusting the levels of PBPs. The temperature dependent expression of PBP2 observed in *clpX* cells, however, indicates that ClpX plays a role in processes related to cell wall synthesis at the lower temperature.

**Fig 2:**
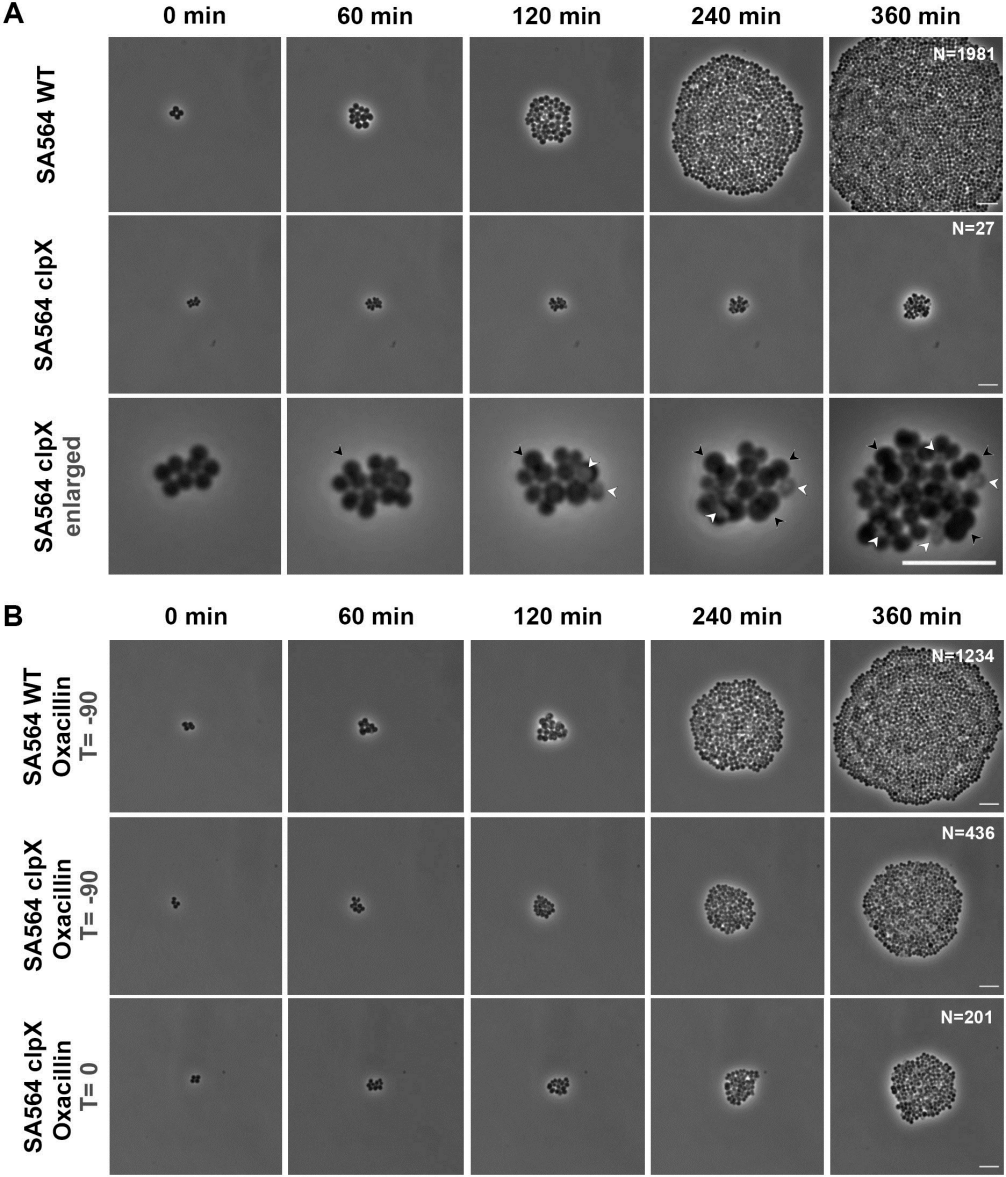
Single cell analysis reveals that oxacillin prevents premature growth arrest and spontaneous lysis of *S*. *aureus clpX* mutants. Still images from time-lapse microscopy (phase contrast) of SA564 wild-type and *clpX* cells growing on a semisolid surface at 30°C, without (**A**) or supplemented with 0.01 μg ml^-1^ oxacillin (**B**). In the upper and middle panel (T = -90) cells were exposed to 0.01 μg ml^-1^ oxacillin 90 min prior to imaging; in the lower panel (T = 0), *clpX* cells were grown in the absence of oxacillin prior to imaging. The still images are taken from movies (see S1 Movie) showing the typical growth of one micro-colony among at least 20 imaged micro colonies, except that the *clpX* cells depicted in (A) belong to the minority of *clpX* cells that were capable of initiating growth and forming a micro colony. N, corresponds to the number of living cells at the endpoint (T = 360). Scale bar, 5 μm.

To further investigate how β-lactams improve growth of the *clpX* cells, we studied growth of single cells of the *S*. *aureus* SA564 wild type and *clpX* mutant in the absence or presence of oxacillin at 30°C using automated phase contrast time-lapse microscopy. The time-lapse experiments revealed that in the absence of oxacillin only about half of the imaged *clpX* cells (15 of 33 cells) were capable of initiating growth and forming micro-colonies that compared to wild-type colonies were of significantly reduced size with an average number of cells of only 47±59 as compared to 1736±384 in wild-type colonies (P<0.0001) (Fig 2A and S1 Movie). The remaining 18 *clpX* cells either did not initiate dividing or stopped dividing early on in the experiment (S1 Movie; black arrows and in Fig 2A). Strikingly, in the *clpX* micro-colonies a large fraction (16 ± 6%) of cells lysed spontaneously during the course of the experiment (S1 Movie; white arrows and in Fig 2A). The cells in Fig 2 were grown at 30°C for 90 min prior to imaging, however, growth arrest and spontaneous lysis could be observed right after shifting *clpX* cells from 37°C to 30°C, demonstrating that a down-shift in temperature has an immediate impact on cells devoid of ClpX activity. Interestingly, oxacillin clearly stimulated growth of *clpX* cells, and when exposed to oxacillin all imaged *clpX* cells (26 of 26 imaged cells) were capable of initiating growth and ended up forming micro-colonies with significantly higher cell numbers than in the absence of oxacillin (351±175, P<0.0001; Fig 2B and S1 Movie). In comparisons, the final cell count reached in wild-type micro-colonies was slightly reduced in the presence of oxacillin (Fig 2 and S1 Movie). Remarkably, oxacillin appears to greatly reduce the number of *clpX* cells undergoing spontaneous lysis (from 16 ± 6% to 4 ± 2%; P<0.0001), and *clpX* cells exposed to oxacillin at the start of imaging (T = 0), initiated growth almost as fast as *clpX* cells that were pre-exposed to oxacillin for 90 min prior to imaging (T = -90). Hence, oxacillin seems to be capable of stimulating growth of *clpX* cells almost immediately. This important finding rules out the contribution of genetic suppressors, and indicates that the binding of oxacillin to the trans-peptidase (TP) site of PBPs per se may be causing the stimulatory effect.

### Aberrant septum synthesis and premature splitting of daughter cells in the *S*. *aureus clpX mutant*

**Fig 3:**
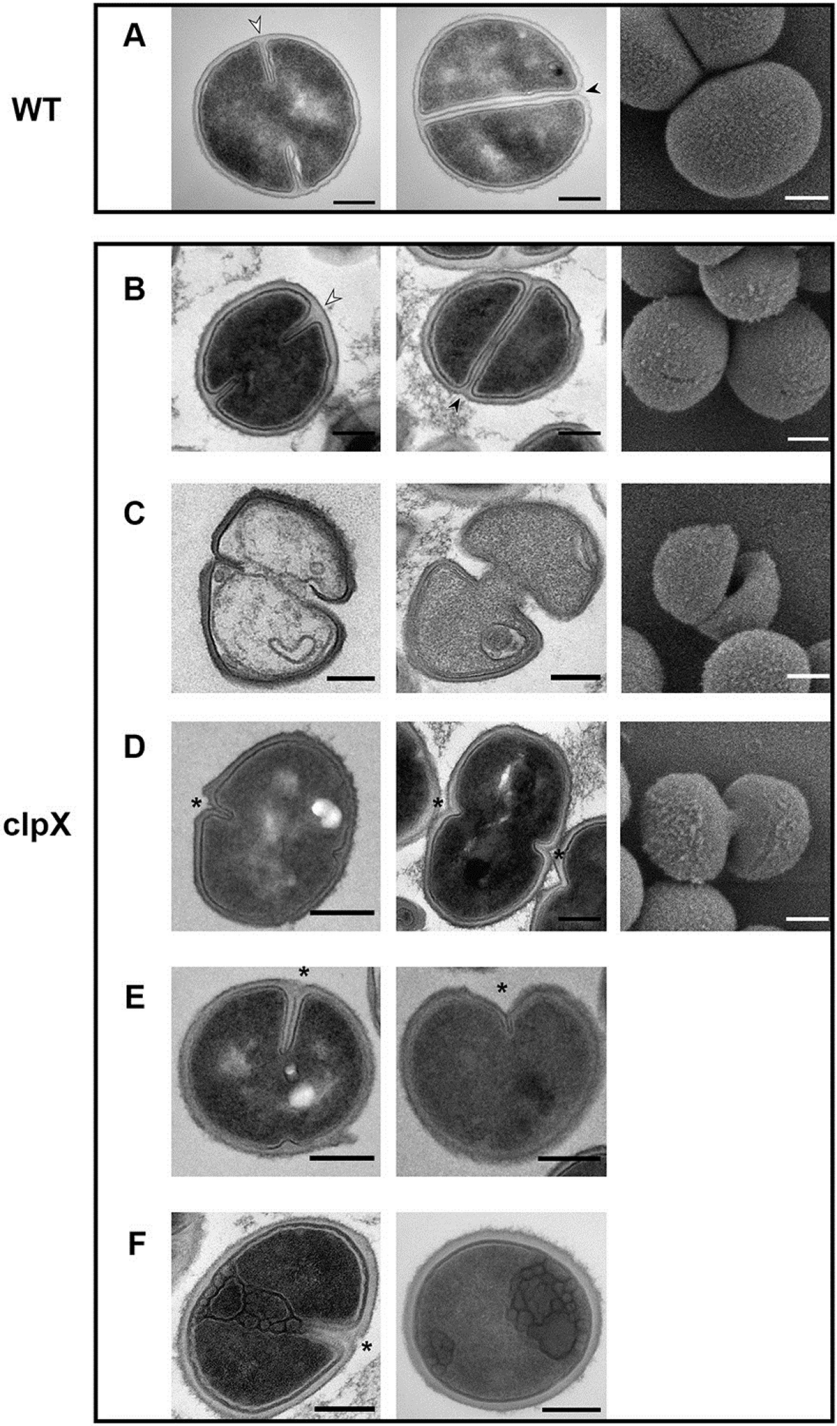
*S*. *aureus clpX* cells grown at 30°C display aberrant septum ingrowth and initiate daughter cell separation prior to septum closure. TEM (left panels) and SEM (right panel) images of SA564 wild-type (**A**) or *clpX* cells (**B-F**) grown in TSB to mid-exponential phase at 30°C. Images show characteristic morphologies of SA564 wild-type or *clpX* cells at 30°C as determined from at least three biological replicates. *clpX* cells displaying the normal coccoid morphology (**B**); the typical appearance of lysed *clpX* cells (**C**); *clpX* cells with non-divided cytoplasma displaying premature splitting of daughter cells (**D**); *clpX* cells displaying asymmetrical septum ingrowth, (**E**); *clpX* cells displaying mesosome-like structrues at the septal site (**F**). White arrows point to the intact peripheral cell wall at the site of septum that is typical for wild-type cells with unclosed septa, while black arrows point to signs of daughter cell splitting initiating from the peripheral wall in cells with closed division septa. Asterisk mark *clpX* cells that despite displaying an incomplete septum show signs of daughter cell splitting initiating from the peripheral wall. The displayed morphological changes are typical for *clpX* cells also in other *S*. *aureus* strain backgrounds tested (8325–4, and JE2). Scale bar, 0.2 μm.

To further investigate the *clpX* phenotypes, we studied the morphology of wild-type and *clpX* cells by transmission electron microscopy (TEM) and scanning electron microscopy (SEM) after growth at 30°C. In general, cells lacking ClpX were smaller than wild-type cells (V = 0.42 ± 0.1 μm^3^ as compared to V = 0.7 ± 0.1 μm^3^, P < 0.001) and have a thickened cell wall (Fig 3A), consistent with previous results described for *clpX* cells growing at 37°C [23]. However, *clpX* cells growing at 30°C displayed a number of distinctive morphological changes that were not observed at 37°C. First, consistent with the spontaneous cell lysis observed in the time-lapse microscopy, approximately 10% of the *clpX* mutant cells grown at 30°C appeared as lysed ghost cells in the TEM images (S3 Fig). Interestingly, these ghost cells had a characteristic appearance in which the cell wall was ripped apart at the tips of the ingrowing, still incomplete, septa (see examples in Fig 3C), indicating that these cells underwent lysis while in the process of daughter-cell splitting. To divide, *S*. *aureus* builds a septal cross wall generating two hemispherical daughter cells connected through a narrow peripheral ring [24,25]. Resolution of this peripheral wall ring leads to rapid splitting of daughter cells, in a process designated as “popping” [25]. Popping has been described to take place only in cells with closed septa and, consistent with this notion, the peripheral wall at the site of septum always appeared intact in wild-type cells displaying incomplete septa (marked with white arrow in Figs 3A and S3), while invaginations/breakage in the peripheral wall at the site of septum were visible only in wild-type displaying a completed septum (marked with black arrow in Figs 3A and S3). In contrast, a substantial fraction of *clpX* cells with still incomplete septa displayed invaginations in the peripheral wall at the edge of the septum, or a complete splitting of the ingrowing septum, indicating that they have initiated daughter cell splitting from the cell periphery (black stars in Figs 3D and 3E and S3). *clpX* cells in the process of splitting despite displaying a non-closed septal cross-wall (seen as a hole in Fig 3C, right panel), or, while still being connected by an undivided cytoplasm could also be observed in SEM-images (Fig 3D, right panel). Taken together, these findings strongly suggest that in the absence of ClpX, the system controlling the onset of autolytic separation of daughter cells becomes dysregulated, and that premature splitting of *clpX* cells with incomplete septa results in cell lysis. TEM-images also showed that some *clpX* cells displaying premature split appeared elongated (as also observed in the time-lapse experiment, Fig 2A), see examples in Fig 3D and 3F, or displayed asymmetrical ingrowth of septa, and in extreme cases extending inwards only from one side (+/- premature split; Fig 3D and 3E). This contrasts with wild-type *S*. *aureus* cells whose septa always extended symmetrically inwards from the edge of the cell wall (Figs 3A and S3). Finally, in some *clpX* cells, unordered membranous material reminiscent of mesosome-like structures [26] were observed at the site of septum ingrowth (Fig 3E and 3F). The latter phenotypes suggest that ClpX also contributes to coordinating septum formation in *S*. *aureus*.

### The ClpX chaperone becomes critical for septum completion at 30°C

**Fig 4:**
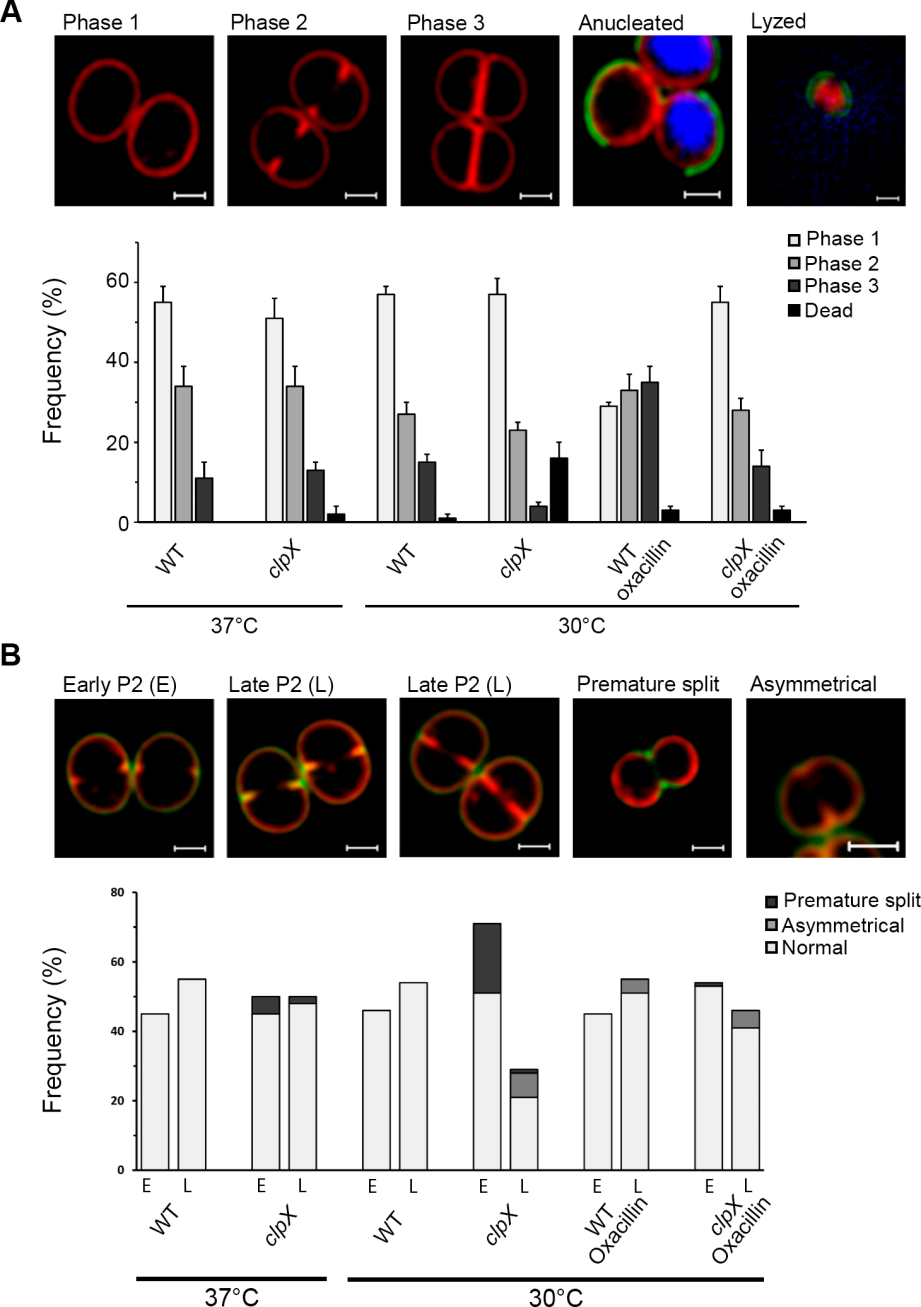
Oxacillin restores progression of the cell cycle in *S*. *aureus clpX* cells grown at 30°C. SA564 wild-type and *clpX* cells were grown at 37°C or 30°C as indicated in the absence or presence of 0.05 μg ml^-1^ oxacillin (**A and B**); cells were then stained with the membrane dye Nile Red (red) and the cell wall dye Van-FL (green) before imaging by SR-SIM. To examine if ClpX alters progression of the growth cycle, 300 cells (from each of two biological replicates) were scored according to the stage of septum ingrowth: no septum (phase 1), incomplete septum (phase 2), or non-separated cells with complete septum (phase 3), according to the examples images shown in the (**A**) top panel. To estimate the fraction of dead cells, the DNA dye Hoechst 3334 was used to identify anucleated and lysed cells–see two examples depicted to the right in the top panel. Scale bar, 0.5 μm. (**B**) Phase 2 cells (200 phase 2 cells for each biological replicate) were additionally scored according to the state of septum ingrowth (cells with less than 15% septum ingrowth were scored as “early” (E), while cells with more than 15% septum ingrowth were scored as “late” (L)), and whether the ingrowth was asymmetrical, as shown in the example images in the top panel. The proportion of cells presenting premature split was estimated based on the Van-FL staining. Scale bar, 0.5 μm.

To accurately quantify morphological phenotypes and to assess the influence of *clpX* on *S*. *aureus* cell cycle progression, we performed Super-Resolution Structured Illumination Microscopy (SR-SIM) on cells stained with the membrane dye Nile red, and scored cells according to the stage of septum ingrowth as described by Monteiro et al. [24]; see Fig 4A for example images. To enumerate cells with incomplete septa that show signs of premature splitting, cells were additionally stained with fluorescently modified vancomycin (Van-FL), which labels the entire cell wall (cell periphery and septum), or with a green fluorescent derivative of wheat germ agglutinin WGA-488 that only labels the peripheral wall [24,27]. To estimate the number of lysed cells, DNA was stained with the blue dye Hoechst 3334. In this analysis, no differences in the distribution of cells in the different phases were observed for wild-type and *clpX* cells grown at 37°C (Fig 4A). At 30°C, however, significantly fewer *clpX* cells displayed a complete septum (phase 3) (4% as opposed to 15% of wild-type cells; P < 0.001). Moreover, while the fraction of cells that were in the process of building a septum (phase 2) was similar in wild-type and *clpX* cells at both temperatures, a more detailed analysis of the phase 2 cells revealed striking differences (Fig 4): consistent with the TEM analysis, a substantial number of *clpX* cells with incomplete septa showed signs of premature daughter cell splitting (20% of phase 2 cells), or had asymmetrical septum ingrowth (7% of phase 2 cells), when cells were grown at 30°C. None of these phenotypes were observed in wild-type cells at any temperature. While asymmetrical septum ingrowth was not observed in *clpX* cells grown at 37°C, premature splitting cells could be observed, however, at a lower frequency (Fig 4B). Furthermore, when subdividing phase 2 cells into two subclasses based on the extent of septum ingrowth, the proportion of *clpX* cells that just started septum ingrowth (defined as cells with less than 15% septum ingrowth; see examples in Fig 4B) was significantly higher (P < 0.001) at 30°C compared to 37°C, and when compared to the wild-type. For wild-type cells, an equal fraction of cells displayed early septum ingrowth at 30°C and 37°C. Finally, SR-SIM confirmed that the fraction of lysed *clpX* cells increased significantly when the temperature was decreased (2% at 37°C, and 16% at 30°C, P < 0.001). In comparison, the proportion of lysed wild-type cells was estimated to be below 2% at both temperatures.

In conclusion, the proportion of *clpX* mutant cells displaying a complete septum or late septum ingrowth was significantly reduced at 30°C, while the proportion of *clpX* cells displaying early septum ingrowth and aberrant septum was significantly increased at 30°C. Thus, the microscopy analyses suggest that ClpX chaperone activity becomes critical for the ability of *S*. *aureus* to complete the division septum as the temperature decreases.

### Oxacillin restores the cell cycle of *clpX* cells

To further examine how β-lactams improve growth of the *clpX* mutant, we performed SR-SIM analysis on oxacillin treated wild-type and *clpX* mutant cells grown at 30°C, as described above (Fig 4). Interestingly, sub-lethal concentrations of oxacillin significantly increased the fraction of phase 3 cells (closed septum): from 15 to 31% in the wild-type (P < 0.001), and from 4 to 14% in the *clpX* mutant (P < 0.001). Moreover, oxacillin significantly decreased the fraction of *clpX* cells (phase 2) that had initiated cell separation prior to septum completion from 20% to 2% (P < 0.001), and in line with this observation, almost no lysed *clpX* mutant cells were observed (Fig 4B). Hence, oxacillin increases the fraction of cells with complete division septa in both the wild-type and the *clpX* backgrounds, and prevents premature splitting of *clpX* cells with incomplete division septa. In contrast, asymmetrical ingrowth of septa is still readily observed in oxacillin treated *clpX* mutant cells (Fig 4A and 4B). These conclusions were supported when the oxacillin treated SA564 wild-type and *clpX* cells were analyzed by TEM; additionally the TEM images indicated that oxacillin prevents formation of mesosome-like structures in *clpX* cells (S4 Fig). Oxacillin treatment, however, conferred a number of well described morphological changes that were shared by wild-type and *clpX* cells including blurring of the electron-dense septal mid-zone, a more fuzzy surface, and thickening of septa. Finally, many daughter cells that have initiated inward splitting from the cell periphery remain incompletely separated at mid-cell (S4 Fig) [7,27,28]. Hence, both the SR-SIM and the TEM images support that oxacillin, even in concentrations well below the MIC value, prolongs phase 3 and delays splitting of the septum in both wt and *clpX* cells.

### Oxacillin antagonizes the septum progression defects conferred by inactivation of ClpX and promotes septal PG synthesis in *clpX* cells with premature split

**Fig 5:**
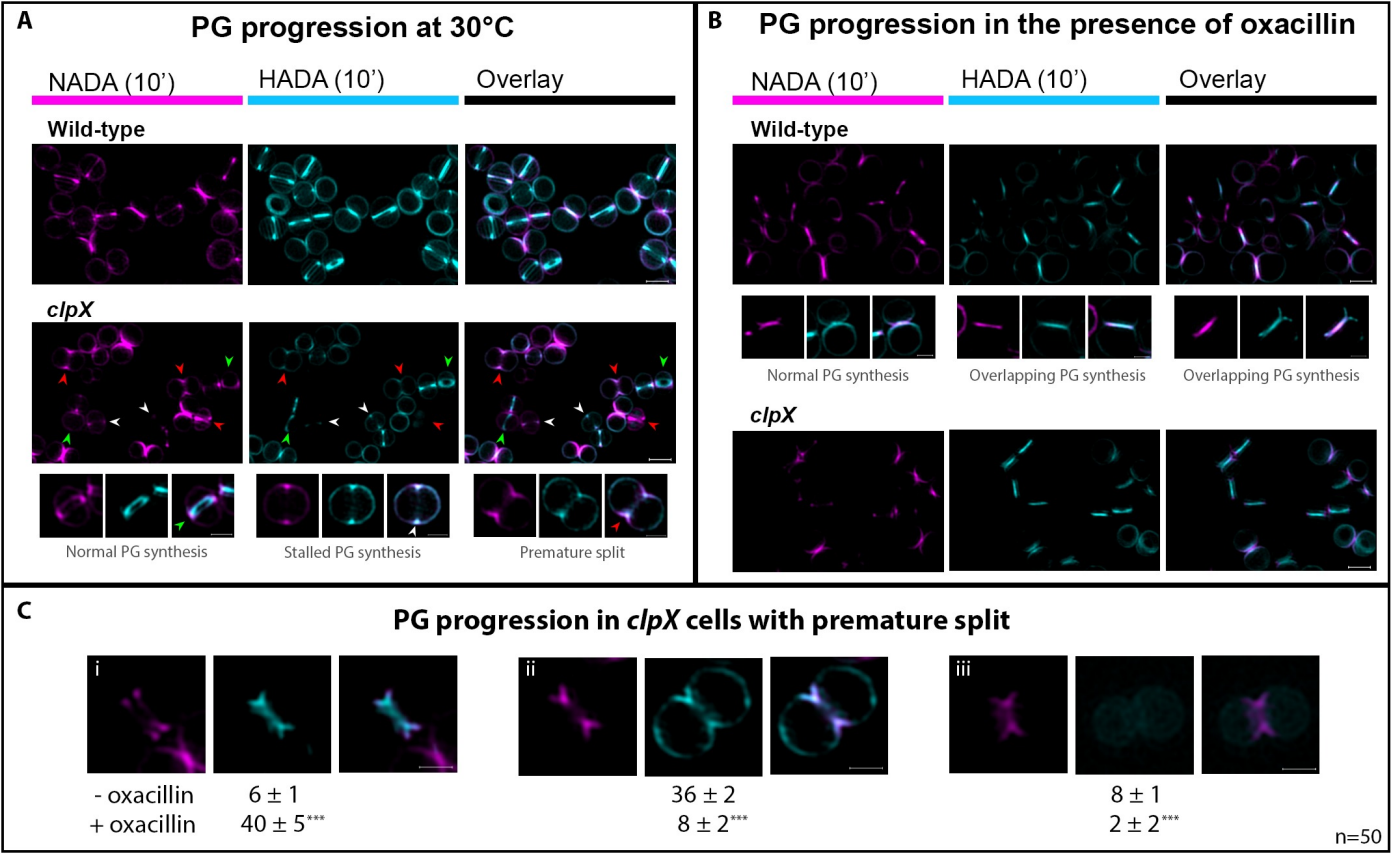
Aberrant progression of septal PG synthesis in *S*. *aureus clpX* cells is rescued by oxacillin. *S*. *aureus* wild-type (SA564) and *clpX* cells were grown at 30°C in the absence (**A** and **C**) or presence of 0.05 μg ml^-1^ oxacillin (**B** and **C**), and PG synthesis was followed by sequentially labeling with NADA for 10 min, followed by washing and labeling with HADA for additional 10 min before SR-SIM imaging. In order to improve contrast, the NADA signal is displayed in magenta, while the HADA signal is displayed in cyan. In the absence of oxacillin (**A**) septal PG synthesis progressed predictably in wild-type cells and in some *clpX* cells (= non-overlapping septal NADA and HADA signals, marked with green arrows). In contrast, some *clpX* cells that initiated septum formation during the first period of labeling showed co-localization of NADA and HADA signals in an early septum ingrowth + HADA signal in the peripheral wall (examples are marked with white arrows), and in some of these cells splitting of the premature septum was observed (examples marked with red arrows). Enlarged examples are depicted in the lower panel. (**B**) In the presence of sub-lethal concentrations of oxacillin, some wild-type cells displayed overlapping NADA and HADA septal signals (examples displayed in middle panel), a phenotype that was not observed for *clpX* cells grown in the presence of oxacillin. (**C**) To examine progression of septal PG synthesis in *clpX* cells displaying premature septal split, 50 cells from each of three biological replicates (grown +/- oxacillin) that initiated septum formation during incubation with NADA, and displayed premature splitting were randomly selected. PG synthesis was followed by assessing HADA incorporation. **(i-iii)** show examples and distribution of the three phenotypes observed. **(i)** show the number of cells where septum synthesis was continued; **(ii)** shows the number of cells that did not continue septum synthesis and instead displayed a HADA signal in the peripheral wall; **(iii)** shows the number of cells where no HADA signal was detected. Numbers are given as the mean and SD of the three biological replicates. Scale bars, 0.5 μm. *** P < 0.001; statistical analysis was performed using the chi square test for independence.

To directly assess the impact of ClpX and oxacillin on progression of septal PG synthesis, we used fluorescent D-amino acids (FDAAs) to visualize regions of new PG insertion [24,29,30]. PG synthesis was followed at 30°C and 37°C by sequentially labeling cells with FDAAs of different colors, thereby creating a virtual time-lapse image of PG synthesis [24,29,30]. Cells were first pulse-labeled for 10 min with green nitrobenzofurazan-amino-D-alanine (NADA), followed by a 10-min pulse with the blue hydroxycoumarin-amino-D-alanine (HADA). Labeled cells were imaged by SR-SIM, and progression of PG synthesis was scored in 300 randomly picked wild-type and *clpX* mutant cells grown in the absence or presence of oxacillin (Fig 5; in order to improve the contrast NADA is displayed in magenta, while HADA is displayed in cyan). In the absence of oxacillin, PG synthesis proceeded from phase 1 (no septa, PG synthesis takes place in the lateral wall) to phase 2 (septal PG synthesis progresses inwards), and finally phase 3 (closed septum, PG synthesis occurs in both septum and the lateral wall) in > 95% of wild-type cells, as described in [24,25] (see Fig 5A). When the *clpX* mutant was grown at 37°C, PG synthesis followed the wild-type paradigm (S5 Fig). In contrast, when the *clpX* mutant was grown at 30°C, the septal PG synthesis progressed abnormally in a substantial fraction of phase 2 cells, as 22 ± 3% of the *clpX* cells that had initiated septum formation in the first period of labeling (NADA) did not continue septum synthesis in the second period of labeling (HADA). Instead, the HADA signal co-localized with the NADA signal in the early septum ingrowth, and additionally, a peripheral HADA signal was visible (marked with white arrows in Fig 5A). Because other *clpX* cells displaying NADA labeling in an early septal ingrowth were indeed capable of septum progression and septum closure (green arrows in Fig 5A), the septal PG synthesis rate does not seem to be generally reduced in the *clpX* mutant. Instead, the co-localization of the NADA and HADA in an early septum ingrowth may reflect stalling of inward septum progression in a subpopulation of *clpX* cells. Interestingly, in the presence of a sub-lethal concentration of oxacillin the fraction of *clpX* cells displaying co-localization of NADA and HADA at the early-septum ingrowth was reduced to 6 ± 2% (Fig 5B).

FDAAs only incorporate into newly synthesized PG and therefore premature splitting initiating from the peripheral wall cannot be detected with this approach [30]. However, splitting of newly synthesized, still incomplete, septum was observed (red arrows in Fig 5A), and while this phenotype was not observed in wild-type cells, this phenotype was displayed in about 20 ± 2% of the *clpX* cells (phase 2 cells) grown in the absence of oxacillin. In the presence of oxacillin, only 8 ± 2% of *clpX* cells showed splitting of newly synthesized still incomplete septa (see example in Fig 5B). In wild-type cells grown in the presence of oxacillin, NADA- and HADA signals more often co-localized in the entire septal plane (examples depicted in Fig 5B), supporting that wild-type cells grown with oxacillin spend longer time in phase 3. We conclude that at temperatures below the optimum, the ClpX chaperone activity becomes important for *S*. *aureus* septal PG synthesis to proceed beyond the point of septum initiation, and that oxacillin antagonizes the septum progression defects conferred by inactivation of ClpX.

The results presented so far suggest that oxacillin improves growth of an *S*. *aureus clpX* mutant by allowing inward progression of the division septum and inhibiting premature splitting and lysis of daughter cells. To investigate septal PG synthesis in cells with premature splitting, we randomly picked 50 *clpX* cells grown at 30°C that had initiated septum formation during incubation with NADA, and that displayed the characteristic morphology of premature splitting, and assessed where HADA was incorporated in these cells. Interestingly, only very few *clpX* cells displaying premature septum split continued synthesizing septum (Fig 5C-i); instead HADA was incorporated at the cell periphery (Fig 5C-ii). In a few cells no HADA signal was detected at all (Fig 5C-iii). Hence, septal PG synthesis seems to stop and instead become dispersed to the peripheral wall in *clpX* cells displaying splitting of a yet incomplete septum. Remarkably, in oxacillin treated cells, septum synthesis progressed normally in most cells with premature split (40 ± 1 of 50 cells, P < 0.001, Fig 5C). Taken together, this analysis demonstrates that oxacillin antagonizes the arrest of septum synthesis observed in *clpX* cells with premature septal split.

### FtsZ localization and constriction of the Z-ring are not affected in the *clpX* mutant

**Fig 6:**
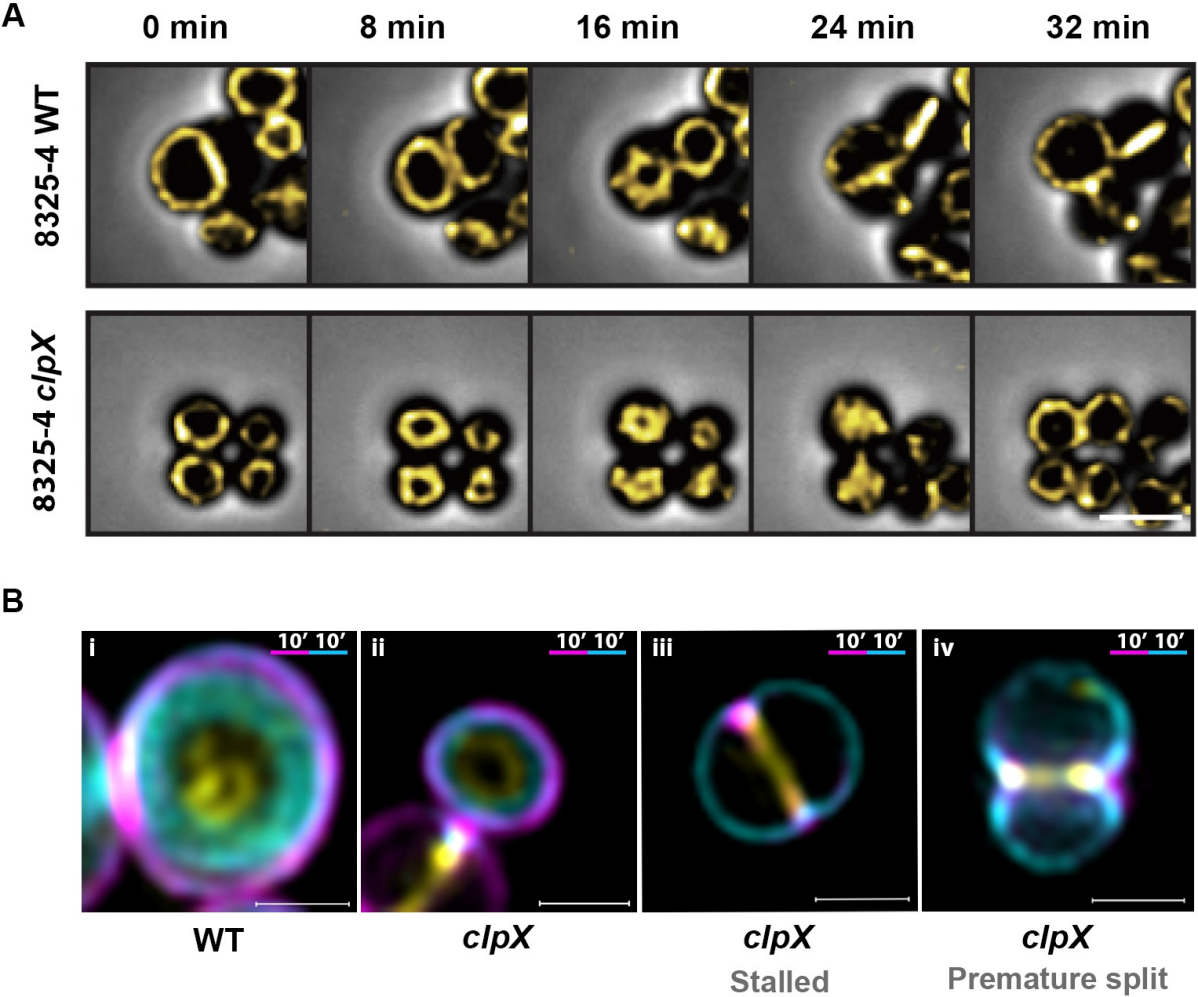
FtsZ localization and Z-ring dynamics appear similar in *S*. *aureus* wild-type and *clpX* mutant cells. FtsZ localization and dynamics were analyzed in *S*. *aureus* wild-type and *clpX* mutant expressing an eYFP-tagged derivative of FtsZ from an IPTG-inducible promoter. (**A**) Still images from time-lapse fluorescence microscopy showing FtsZ-eYFP dynamics in *S*. *aureus* wild type and *clpX* cells growing on a semi-solid matrix in the presence of 100 uM IPTG at 30°C; (S2 Movie). Images are shown as overlay of phase contrast and the YFP signal, scale bar 1 μm. (**B**) The FtsZ-eYFP signal localizes ahead of the site of active PG synthesis: localization of FtsZ relative to PG synthesis was analyzed by sequentially labeling *S*. *aureus* wild-type and *clpX* cells growing in TSB + 50 uM IPTG at 30°C with TADA (red, but displayed in magenta) for 10 minutes followed by washing and labeling with HADA (blue, but displayed in cyan) for 10 min prior to SR-SIM imaging. **(i-iv)** Examples of cells that started septum synthesis in the first period of labeling: wild-type cell (**i**); *clpX* cell with normal progression of PG synthesis (**ii**); *clpX* cell with stalled septum synthesis (HADA-signal localizes at the peripheral cell wall) (**iii**); *clpX* cell displaying premature split (**iv**). Overview images can be found in S6 Fig. Scale bars 0.5 μm.

ClpX from diverse bacteria interacts directly with FtsZ suggesting that the ClpX chaperone has a conserved role in assisting assembly/disassembly of the FtsZ polymer [31–34]. We therefore reasoned that ClpX may regulate septum progression by interfering with FtsZ. To study localization and constriction of the FtsZ-ring, a plasmid expressing eYFP-tagged derivative of FtsZ from an IPTG-inducible promoter [35] was introduced into the SA564 *clpX* mutant. However, although we succeeded in introducing the FtsZ::eYFP plasmid into the SA564 *clpX* mutant in several occasions, the fluorescent signal was lost upon further cultivation of the strain, suggesting that the expression of FtsZ::eYFP becomes toxic to SA564 devoid of ClpX even at 37°C. In contrast, the plasmid could be stably maintained in the 8325–4 background, and FtsZ localization and dynamics were instead performed in this strain. In both wild-type and *clpX* mutant cells, the Z-ring changed predictably throughout the cell cycle (S2 Movie and Fig 6A): in newly divided cells, the Z ring has the same diameter as the cell until the ring starts to constrict and eventually closes (as described in [36]). Following closure, FtsZ undergoes a period of highly dynamic re-distribution, before the Z-ring cycle starts over again in newly divided cells. Hence, FtsZ dynamics appear not to be affected by lack of ClpX activity. Next, we imaged the relative localization of FtsZ and PG synthesis by sequentially labeling PG synthesis with FDAAs as described above, except that tetramethylrhodamine 3-amino–d-alanine (TADA, red signal but displayed in magenta) was used instead of NADA to avoid overlap with the yellow eYFP signal. In both wild-type and *clpX* cells, the eYFP signal localized ahead of septal PG synthesis in all phase 2 cells (Fig 6B and overview in S6 Fig). Specifically, FtsZ also localized ahead of the FDAA signal in *clpX* cells having HADA and TADA signal co-localizing in an early septum in growth (Fig 6B). Strikingly, the FtsZ signal maintained its septal localization in *clpX* cells with premature split and arrest of septal PG-synthesis (see example in Fig 6B). As also shown above, PG incorporation in such cells takes place in the peripheral wall. Hence, our data supports the idea that FtsZ dynamics is not impeded in cells lacking ClpX.

### The TarO inhibitors, tunicamycin and tarocin A1 also rescue growth of the *clpX* mutant

**Fig 7:**
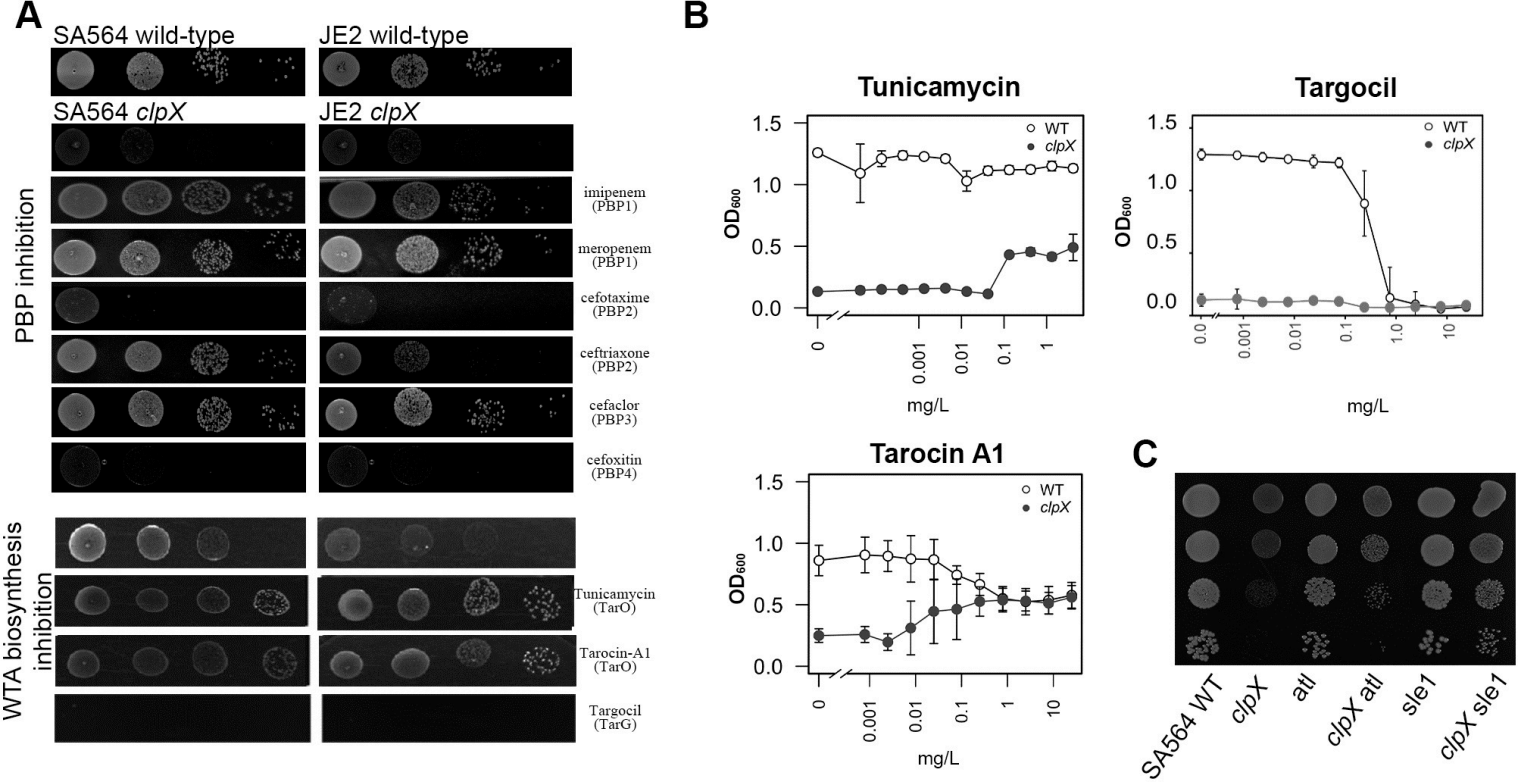
β-lactam antibiotics targeting PBP1or BPB3, and WTA (TarO) inhibitors specifically promote growth of the *clpX* mutant. (**A)** The *S*. *aureus* wild-type strains, SA564 (MSSA) and USA300 JE2 (MRSA) and the *clpX* deletion strains derived here from were grown exponentially in TSB at 37°C. At OD_600_ = 0.5, cultures were diluted 10^1^, 10^2^, 10^3^ and 10^4^-fold, and 10 μl of each dilution was spotted on TSA plates in the presence or absence of subinhibitory concentrations (1/5 MIC of wild-type) of β-lactams with different PBP specificities as indicated; the TarO inhibitors tunicamycin (0.5 ug/ml), and tarocin A1 (0.5 ug/ml), or the TarG inhibitor, targocil (0.2 ug/ml), and incubated at 30°C for 24 h. (**B**) *S*. *aureus* SA564 wild type and the *clpX* deletion strains were grown overnight at 37°C, diluted 1:200 and grown at 37°C until mid-exponential phase. These cultures were then diluted into TSB alone (control) or containing increasing concentrations of tunicamycin, tarocin A or targocil in a 96-well format, and the plates were incubated for 24 h at 30°C. The average final OD and standard deviations from three biological replicates were plotted. (**C**) The *S*. *aureus* SA564 wild-type strain, and the indicated mutant strains were grown exponentially in TSB at 37°C. At OD_600_ = 0.5, cultures were diluted 10^1^, 10^2^, 10^3^ and 10^4^-fold, and 10 μl of each dilution was spotted on TSA plates and incubated at 30°C for 24 h.

Next, we asked if the ability to rescue growth of a *S*. *aureus clpX* cells is specific for the β-lactam class of antibiotics, and whether it depends on inhibition of specific PBPs (S7 Fig). The compounds assessed were either antibiotics with completely different targets, compounds inhibiting various steps in the cell envelope synthesis pathway, or β-lactams that inhibit the four *S*. *aureus* PBPs with different specificities [37–40]. Intriguingly, tunicamycin and tarocin A1, two well characterized inhibitors of the first step in the WTA biosynthesis pathway that work synergistically with β-lactams to kill MRSA [14,16], were the only non β-lactam compounds that stimulated growth of the *clpX* mutant (Figs 7A and 7B and S7). In contrast, targocil that inhibits a late step in WTA biosynthesis, and does not restore sensitivity of MRSA to β-lactams [41], did not improve growth of the *clpX* mutant (Figs 7A and 7B and S7). Similarly, late stage inhibitors of PG synthesis, such as vancomycin and lysostaphin that interfere with PG-crosslinking (vancomycin through binding to the d-Ala-d-Ala dipeptide PG-stem unit, and lysostaphin, which breaks already formed cross-bridges), did not stimulate growth of the *clpX* mutant (S7 Fig). Taken together, these findings demonstrate that neither inhibition of WTA synthesis nor reducing PG cross-linking *per se* will alleviate the growth defect of the *clpX* mutant. Testing β-lactams with varying PBP specificities showed that β-lactams specifically inhibiting PBP1 (meropenem, imipenem, and cloxacillin), or PBP3 (cefaclor) stimulated growth of the *clpX* mutant most efficiently (Figs 7A and S7). Therefore, the specific binding of β-lactams to the trans-peptidase (TP) domain of PBP1 and PBP3 seems to be crucial for the ability of β-lactams to antagonize the severe growth defect imposed by the lack of ClpX at 30°C.

### Inactivation of the Sle1 autolysin rescues growth of *clpX* cells

The findings that tunicamycin, tarocin A1, and β-lactam antibiotics specifically rescue growth of the *clpX* mutant point to the existence of a functional link between the early steps of WTA biosynthesis and the TP domain of PBPs that is critical for alleviating the *clpX*-phenotype. LTA synthesis seems to play a part in the same process, as loss-of-function mutations in *ltaS* also rescue growth of the *clpX* mutant [20]. Of special interest to the present study, LTA and ClpX have opposite roles in determining the level of the two major autolysins involved in daughter cell splitting, namely Sle1 and Atl [20]. One possible scenario is therefore that the elevated levels of Atl and Sle1 autolysins are causing premature splitting and lysis of *clpX* cells unless localization or activation of these autolysins is prevented. This model would be consistent with earlier reports demonstrating that WTA and LTA promote septal localization of autolysins [14,20,42,43], and with our finding that oxacillin delays splitting of cells with completed septa. In support that autolysins contribute to the growth defect of the *clpX* mutant at 30°C, we found that inactivation of *sle1*, and to a minor extent inactivation of *atl*, enabled the *clpX* strain to form visible colonies at 30°C (Fig 7C). Thus, one simple scenario would be that β-lactam antibiotics and TarO inhibitors rescue growth of the *clpX* mutant by antagonizing mainly Sle1-mediated lysis.

## Discussion

Because mis-coordination in activation of autolytic enzymes may have fatal consequences, regulatory checkpoints that coordinate the autolytic system with septum completion likely exist, however, little is known about these mechanisms. Here, we show that the widely conserved ClpX chaperone plays a temperature dependent role in staphylococcal cell division resulting in severe morphological changes at 30°C but not at 37°C. In wild-type *S*. *aureus* cells, splitting of daughter cells is not initiated prior to septum closure. In contrast, a substantial fraction of *clpX* cells displaying incomplete septa had initiated splitting of daughter cells indicating that the system responsible for coordinating autolytic splitting with septum completion has become dysregulated. In *clpX* cells displaying the premature splitting phenotype, septal PG synthesis did not progress inwards, demonstrating that *clpX* cells with premature split are unable to finalize the septum. The detrimental character of this defect likely prevents cells from undergoing further divisions, explaining why a large proportion of *clpX* cells are non-dividing and end up lysing. In support hereof, TEM pictures show that most *clpX* ghost cells were in the process of splitting despite having an incomplete septum. This is likely due to turgor pressure forces breaking the tip of the ingrowing septum where the cell wall is thin and mechanically weak [44]. Hence, we assume that premature splitting is the underlying cause for the high rate of spontaneous lysis observed among *clpX* cells.

**Fig 8:**
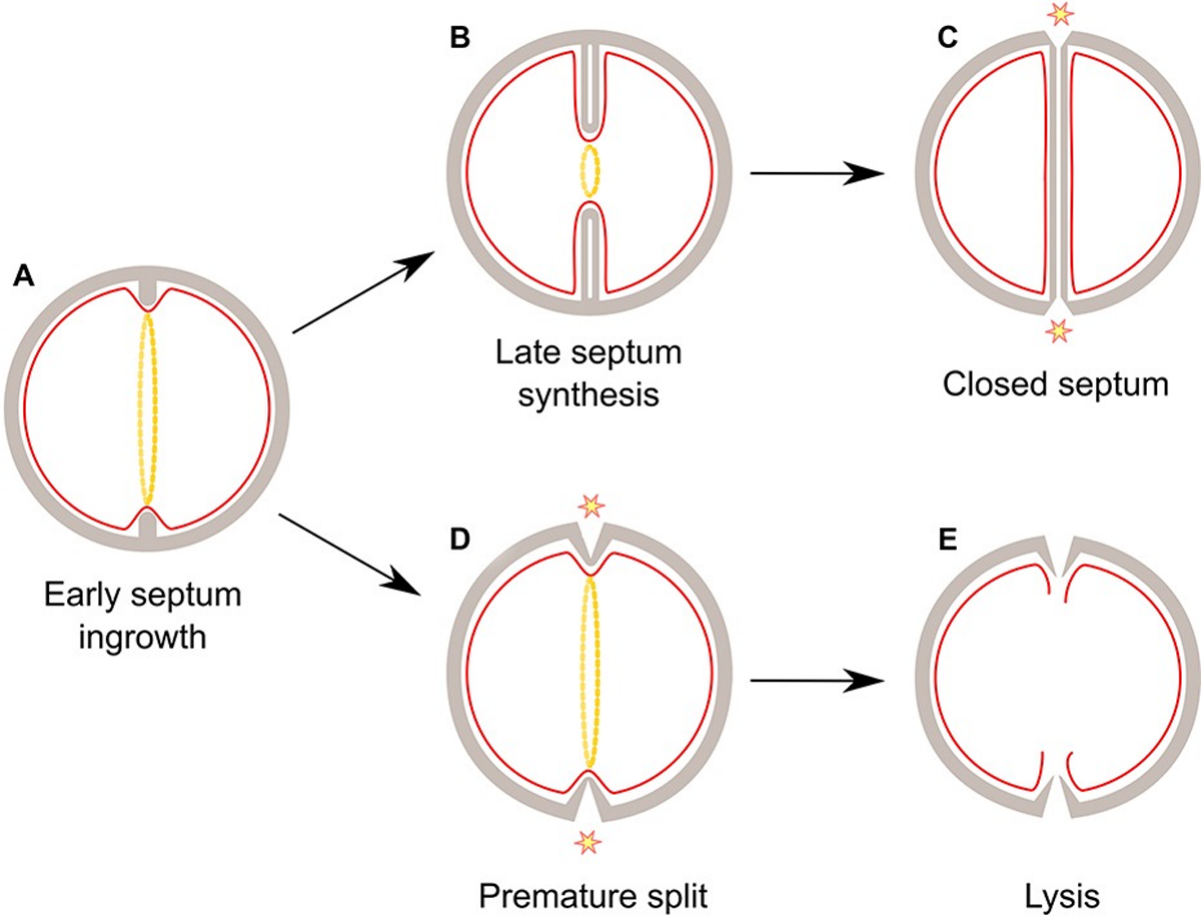
Model of temperature dependent lysis of *S*. *aureus clpX* mutant. At 37°C (upper panel), progression from an early **(A)** to a late septal ingrowth **(B)** occurs in the absence of ClpX activity. Upon septum closure, release of cross-linking substrates from the TP domain of PBPs in combination with teichoic acid biosynthesis serve as signals to activate autolytic splitting of daughter cells (**C**). At 30°C (lower panel), progression from an early to a late septal ingrowth becomes more dependent on assistance from the ClpX chaperone, and in the absence of ClpX, septum synthesis occasionally stalls in an early septal ingrowth that put the cells at risk for activation of Sle1- mediated autolytic splitting from the peripheral wall (**D**). Cells with premature splitting will be prone to lysis due to the risk of turgor pressure forces breaking the thin and mechanically weak cell wall at the tip of the ingrowing septum (**E**).

Importantly, cells devoid of ClpX contain elevated levels of the two major autolysins associated with separation of *S*. *aureus* daughter cells, Sle1 and Atl [20,21,45–47]. Therefore, premature splitting of *clpX* cells could simply be a consequence of excess autolysins, and consistent with this assumption inactivation of *sle1* and to a minor extent *atl* improved growth of the *clpX* mutant at 30°C. However, whilst SleI certainly contributes to the premature splitting and spontaneous lysis of *clpX* cells, additional factors are likely in play, as premature splitting and lysis of *clpX* cells is more frequent at 30°C than at 37°C, despite the finding that autolysin levels are elevated at both temperatures [20,21]. As a halt in inward progression of septum synthesis was observed in *clpX* cells only at the lower temperature, we speculate that this stalling of septum synthesis put the cells at risk for premature activation of autolysins, as depicted in the working model (Fig 8). In this model, *S*. *aureus* depends on ClpX chaperone activity for transforming an early stage divisome complex into a late stage divisome complex at 30°C, but not at 37°C. At both temperatures, the high levels of autolysins will make the *clpX* cells more prone to initiate daughter cell separation before septum completion. However, stalling of the divisome exacerbates the risk of premature split at 30°C. Consistent with this model, premature split could be observed in *clpX* cells grown at 37°C, however, at this temperature septal progression seems to proceed fast enough to enable completion of the septum, as outlined in Fig 8. FtsZ localization and dynamics were not affected in the absence of ClpX, suggesting that ClpX affects septum formation downstream of Z-ring formation. Importantly, cytokinesis in *Bacill*us *subtilis* and *S*. *aureus* is proposed to occur in two-steps: an initial FtsZ dependent slow step that may drive the initial membrane invagination, and a second faster step driven by PG synthesis and recruitment of late division proteins such as PBPs [36,48]. Hence, we speculate that ClpX promotes septum progression at 30°C by directly, or indirectly, assisting assembly of this late divisome complex. Technically this will, however, be challenging to prove, as molecular chaperones like ClpX associate only transiently with folding intermediates of substrate proteins.

Remarkably, the growth and lysis defect imposed by the *clpX* deletion was alleviated by sub-lethal concentrations of β-lactam antibiotics. This intriguing finding is to our knowledge the first example of β-lactam antibiotics being able to promote growth and preventing spontaneous lysis of a bacterial mutant. The presented data show that oxacillin simultaneously rescues septum synthesis, and prevents premature splitting, mesosome formation, and spontaneous lysis of the *clpX* mutant, lending support to a linkage between these phenotypes. The ability of sub-lethal concentrations of β-lactam antibiotics to suppress spontaneous lysis of *clpX* mutant cells was surprising, as loss of wall integrity accompanied by cell lysis is believed to contribute to the lethal activity of β-lactam antibiotics [5,9,49]. Here, we observed that oxacillin treatment of both *S*. *aureus* wild-type and *clpX* mutant cells increased the fraction of cells displaying a complete division septum, supporting previous findings that β-lactams delay autolytic splitting of daughter cells [7,28]. Moreover, the sequential PG staining experiments showed that late septal FDAA signals often overlap in wild-type cells grown in the presence of oxacillin, indicating that β-lactams prolong PG synthesis in the completed septum. Consistent with these findings, β-lactam treated *S*. *aureus* cells display characteristic thickened septum in TEM images [7,28]. Taken together, these findings indicate that the irreversible binding of β-lactams (mimicking substrate binding) to the TP domain of PBPs impedes activation of septal autolysins and abrogates the normal release of PBPs from the septal PG synthesis complex upon completion of the division septum. Hence, we speculate that the unoccupied TP domain upon completion of PG crosslinking plays a role in signaling that PG synthesis is complete, and that it is time to activate septal autolysins, and to release PBPs from the septal site. This hypothesis would be consistent with previous findings indicating that i) the transpeptidation substrates recruit PBP2 to the division site, and that ii) the TP site of PBP1 takes part in a checkpoint-type mechanism ensuring that autolytic splitting of daughter cells can only take place upon completion of septum synthesis [50,51]. Hence, oxacillin may rescue septum synthesis in *clpX* cells with premature split by stabilizing the late septal PG synthesis complex thereby reducing the risk of lysis (Fig 8).

Previously, we showed that the fast-growing suppressor mutants arising when *clpX* cells are grown at 30°C have lost the ability to synthesize LTA [20]. Interestingly, we here show that inhibitors of the first step of WTA synthesis are the only other compounds that similarly to β-lactams rescue growth of the *clpX* mutant. LTA and WTA are both described to be critical for maintaining normal levels of peptidoglycan hydrolase activity [14,42,43], and consistent with these findings the elevated levels of surface-anchored Atl and Sle1 in *clpX* cells are reverted to wild-type levels by inactivation of LtaS [20]. We, hence, speculate that inhibition of teichoic acid synthesis stimulates growth of the *clpX* mutant by antagonizing premature autolytic splitting of daughter cells (Fig 8). To follow up on the finding that TarO inhibitors specifically rescue growth of *clpX* mutants, we asked if genetic inactivation of *tarO* would also rescue growth of the *clpX* mutants, but did not succeed in deleting *tarO* in SA564 and JE2 *clpX* strains. This finding supports that TarO inhibitors do not simply rescue growth of *clpX* mutants by reducing WTA synthesis. Instead we speculate that the binding of tunicamycin or tarocin A1 to the TarO enzyme may, similarly to the binding of oxacillin to PBPs, induce a conformational change that is responsible for the stimulatory effect. However, more experimental data is required to clarify the underlying mechanism.

In conclusion, we have shown that *S*. *aureus* cell division is temperature sensitive, and that the ClpX chaperone serves an important function in coordinating initiation of daughter cell separation with septum completion at 30°C. When ClpX is absent, cell division frequently has a fatal outcome because septal PG synthesis stalls and cell separation is initiated prior to completion of the septum. Interestingly, these defects were prevented by binding of β-lactam antibiotics to the PBP transpeptidase activity domain, indicating that this final stage in PG biosynthesis plays a role in coordinating septum synthesis and activation of autolytic splitting of daughter cells. Our work therefore supports the idea that in this clinically important bacterium, the effect of β-lactam antibiotics is tightly linked to coordination of cell division.

## Materials and methods

### Bacterial strains and growth conditions

Strains used in this study are listed in S1 Table. *S*. *aureus* strains were grown in tryptic soy broth media (TSB; Oxoid) under vigorous agitation at 200 rpm at 37°C. In most experiments, 20 ml of medium was inoculated in 200-ml flasks to allow efficient aeration of the medium. For solid medium, 1.5% agar was added to make TSA plates. Erythromycin (7.5 μg ml^-1^) was added as required. Upon receipt of the low-passage isolate SA564, the strain was cultured once and stored frozen at -80°C. In all experiments, we used bacterial strains freshly streaked from the frozen stocks on TSA plates with antibiotics added as required and incubated overnight at 37°C. The growth was followed by measuring the optical densities at 600 nm. The starting OD was always below 0.05. When inoculating *S*. *aureus clpX* deletion strains, care was taken to avoid visibly larger colonies containing potential suppressor mutants [20]. To minimize the risk of selecting for fast-growing suppressor mutants in broth cultures of *clpX* mutant cells grown at 30°C, strains were first grown at 37°C for four generations (OD_600_ ~0.1–0.2) before shifting to 30°C. *S*. *aureus* JE2-derived strains were obtained from the Network of Antimicrobial Resistance in *Staphylococcus aureus* (NARSA) program (supported under NIAID/NIH contract HHSN272200700055C).

### Growth rate and final yield

All exponential growth rates were determined by growing the relevant strains in a Bioscreen C instrument: For growth in the BioscreenC instrument, overnight cultures were diluted in 300 μl TSB (with or without antibiotics as indicated) to an OD_600_ of approx. 0.001, Plates were incubated at 30°C or 37°C and OD_600_ was measured every 5 min with 20 seconds of shaking before each measurement. The growth rates were automatically calculated as described before [20]. In short, OD_600_ values were log-transformed and linear regressions were determined for each data point in the OD_600_ interval from 0.02 to 0.12 based on a window containing 15 data points. The exponential growth rate was identified as the maximal slope of the linear regressions. The standard error of the mean was calculated using values from three biological replicates. Statistical significance was calculated using Student’s t-test. For end-point ODs, overnight cultures were diluted 1:200 in TSB and grown to exponential phase (OD_600_ 0.1) and then diluted 1:10,000 in 200 μl TSB (with or without antibiotics as indicated) in a 96-well microtiter plate, and incubated 24 h at 30°C with shaking. The final yield was determined by measuring the OD_600_ and by determining cfu ml^-1^ by plate counting.

### Disc diffusion assays

*S*. *aureus* strains were inoculated on TSA plates and incubated at 37°C overnight. The next day, a bacterial suspension was adjusted to a 0.5 McFarland (Sensititre® nephelometer and the Sensititre® McFarland Standard) and streaked on MHA. The plates were allowed to dry prior to the addition of 1 μg oxacillin discs (Oxoid) and incubated at 37°C for 48 hours.

### Time-lapse microscopy

Cultures of SA564 wild-type and SA564Δ*clpX* were grown in TSB at 37˚C for four generations (OD_600_ = 0.1) before shifting cultures to 30˚C and continuing growth in the absence or presence of 0.01 μg ml^-1^ oxacillin (T = -90) for 90 minutes prior to imaging (reaching OD_600_ of 0.5 ± 0.1). Cells were subsequently washed in fresh TSB before spotting on TSB-polyacrylamide (10%) slides supplemented with 0.008 μg ml^-1^ oxacillin when appropriate. Acrylamide pads were placed inside a Gene frame (Thermo Fisher Scientific) and sealed with a coverslip as described before [52]. Phase contrast image acquisition was performed using a DV Elite microscope (GE healthcare) with a sCMOS (PCO) camera with a 100x oil-immersion objective. Images were acquired with 200 ms exposure time every 6 minutes for at least 6 h at 30°C using Softworx (Applied Precision) software. Images were analyzed using Fiji (http://fiji.sc). Each experiment was performed at least in triplicate. 25 micro colonies were imaged for SA564; 27 micro colonies for SA564 + oxacillin; 33 micro colonies for SA564 *clpX;* 24 micro colonies for SA564 *clpX +* oxacillin (T = -90) and 26 micro colonies for SA564 *clpX +* oxacillin with no preexposure (T = 0). The frequency of *clpX* cells undergoing spontaneous lysis was determined by following the fate of all single cells in observable in the 26 and 33 imaged *clpX* micro colonies (grown +/- oxacillin, respectively) throughout the course of the experiment.

To analyze FtsZ localization and dynamics, *S*. *aureus* wild-type (8325-4/pCQ11ftsZ::eYFP) and *clpX* mutant (8325–4ΔclpX/pCQ11ftsZ::eYFP) were grown overnight in TSB medium at 37°C and cultures were diluted 100 times in fresh TSB medium and grown until an OD_600_ of 0.1. Cells were washed once in fresh TSB medium and spotted onto a TSB-polyacrylamide (10%) slide incubated with TSB medium supplemented when appropriate with 100 μM IPTG. Acrylamide pads were placed inside a Gene frame (Thermo Fisher Scientific) and sealed with a cover glass as described before [52].Time-lapse images of FtsZ-eYFP were acquired using a Leica DMi8 microscope with a sCMOS DFC9000 (Leica) camera with a 100x oil-immersion objective and a Spectra X (Lumencor) illumination module. Fluorescent images were acquired every 4 min with 400 ms exposure using a YFP filter cube (Chroma, excitation 492–514 nm, dichroic 520 nm, emission 520–550 nm). Images were processed using LAS X (Leica) and signal was deconvolved using Huygens (SVI) software.

### Muropeptide analysis by HPLC

*S*. *aureus* strains were grown in TSB at 37° until an OD_600_ of 0.1. At this point the cultures were split in two and growth was continued at 30°C in the presence or absence of oxacillin (0.02 ug/Ml) until the OD_600_ reached 0.9. Muropeptides were obtained from purified peptidoglycan digested with the muramidase mutanolysin M1 (Sigma), an *n*-acetylmuramidase that cuts glycan strands between the *n*-acetylmuramic and *n*-acetylglucosamine residues of both O-acetylated and unmodified peptidoglycan, as previously described in [53]. The resulting muropeptides were reduced with sodium borohydride (Sigma) and analyzed by reversed-phase HPLC using a HypersilODS column (Thermo Fisher Scientific, Waltham, MA). Muropeptide species were eluted in 0.1 M sodium phosphate, pH 2.0, with a gradient of 5–30% methanol for 155 min and detected at 206 nm. The eluted muropeptides were detected by determination of their UV absorption at 206 nm, using the software LC SOLUTION (Shimadzu, Kyoto, Japan). Peaks corresponding to monomers, dimers, trimers to higher oligomers were assigned according to previous nomenclature [54].

### PBP profiles using Bocillin-FL and PBP4 Western blot analysis

PBP levels were analyzed as described previously [55]. In short, membrane proteins were purified from late-exponential cultures (OD_600_ of 1) of SA564 wild-type and the corresponding *clpX* mutant grown at 30°C or 37°C in the presence or absence of oxacillin at 0.05 ug/mL. Cells were resuspended in 50 mM Tris, 150 mM NaCl, 5 mM MgCl2 buffer, pH 7.5 supplemented with phenylmethylsulfonyl fluoride (0.5 mM), b-mercaptoethanol (10 mM), Lysostaphin (100 mg ml^-1^), DNase (20 mg/ml, and RNase (10 mg ml^-1^). The cell suspension was incubated at 37°C for 30 min followed by sonication for 5 cycles of 1 min with 2 min intervals on ice between each cycle. The membranes were harvested by ultracentrifugation at 110,000 X g for 40 min at 4°C and solubilized in 2% Triton X-100. 100 ug purified membrane was labeled with Bocillin-FL (100 uM) for 10 minutes at 30°C. The reaction was stopped by adding 5X volume of sample buffer. PBPs were separated on a 7.5% SDS gel and visualized using fluorography. PBP4 levels were additionally determined by Western blot analysis using antibodies specific for *S*. *aureus* PBP4 as described in [21]. Densitometry analysis for three biological replicates was performed using the Fiji “Gel Analysis tool”, where the gel background was removed individually for each band.

### Electron microscopy and image analysis

#### Transmission electron microscopy (TEM)

*S*. *aureus* overnight cultures grown at 37°C were diluted 1:200 into 40 ml of TSB and grown at 30°C or 37°C to an OD_600_ of 0.5. Bacteria cells were collected from a 10-ml aliquot by centrifugation at 8,000 x g, and the cell pellets were suspended in fixation solution (2.5% glutaraldehyde in 0.1 M cacodylate buffer [pH 7.4]) and incubated overnight at 4°C. The fixed cells were further treated with 2% osmium tetroxide, followed by 0.25% uranyl acetate for contrast enhancement. The pellets were dehydrated in increasing concentrations of ethanol, followed by pure propylene oxide, and then embedded in Epon resin. Thin sections for electron microscopy were stained with lead citrate and observed in a Philips CM100 BioTWIN transmission electron microscope fitted with an Olympus Veleta camera with a resolution of 2,048 by 2,048 pixels. Sample processing and microscopy were performed at the Core Facility for Integrated Microscopy (CFIM), Faculty of Health and Medical Sciences, University of Copenhagen.

#### Scanning electron microscopy (SEM)

Exponentially growing *S*. *aureus* cells were collected by centrifugation, fixed in 2% glutaraldehyde in 0.05 M sodium phosphate buffer, pH 7.4 and sedimented on coverslips for 1 week at 4°C. The cells were washed three times in 0.15 M sodium phosphate buffer, pH 7.4 and and specimens post fixed in 1% OsO_4_ in 0.12 M sodium cacodylate buffer, pH 7.4 for two hours. Following a rinse in distilled water, the specimens were progressively dehydrated to 100% ethanol and critical point dried (Balzers CPD 030) with CO_2_. Cells were subsequently mounted on stubs using colloidal silver as an adhesive and sputter coated with 6 nm gold (Leica Coater ACE 200) before imaging with a FEI Quanta 3D scanning electron microscope operated at an accelerating voltage of 2 kV. Sample preparation and SEM imaging was performed at CFIM.

### SR-SIM analysis

SR-SIM was performed with an Elyra PS.1 microscope (Zeiss) using a Plan-Apochromat 63x/1.4 oil DIC M27 objective and a Pco.edge 5.5 camera. Images were acquired with five grid rotations and reconstructed using ZEN software (black edition, 2012, version 8.1.0.484) based on a structured illumination algorithm, using synthetic, channel specific optical transfer functions and noise filter settings ranging from -6 to -8. Laser specifications can be seen in S2 Table. SR-SIM was performed at CFIM.

### Estimation of cell size

The volume of 100 phase 1 cells was determined (three biological replicates) as described in [24]. Briefly, an ellipse was fitted to the border limits of the membrane and measurements of the minor and major axis were acquired. The shape of the cells was assumed to be that of a prolate spheroid and the volume was estimated by the equation V = 4/3πab^2^; a and b correspond to the major and minor axes, respectively. Ellipse fitting and measurements were performed using ImageJ.

### Analysis of the cell cycle

To address progression of the cell cycle, exponential cultures of *S*. *aureus* were incubated for 5 min at room temperature with the membrane dye Nile Red, the cell wall dye WGA-488 or Van-Fl and the DNA dye Hoechst 3334 (S3 Table). Samples were placed on an agarose pad (1.2% in PBS) and visualized by SR-SIM as described above. 300 cells were scored according to the stage of septum ingrowth: no septum (phase 1), incomplete septum (phase 2), or non-separated cells with complete septum (phase 3). Dead cells were scored based on Hoechst staining: lysed cells, as cells where DNA had leaked out of the cell and anucleated cells as cells devoid of Hoechst staining. The analysis was performed on two biological replicates. Additionally, 200 cells were scored according to the state of septum ingrowth by measuring the length of the ingrowing septum relative to the cell diameter using Fiji. Cells with less than 15% septum ingrowth were scored as “early”, while cells with more than 15% septum ingrowth were scored as “late”. Additionally, the fractions of cells displaying asymmetrical septum ingrowth, or showing signs of premature splitting was (based on staining with Van-FL) were scored. This analysis was performed on two biological replicates.

### Analysis of PG synthesis progression

To evaluate localization of PG synthesis, exponential cultures of *S*. *aureus* (SA564 or 8325–4) were pulse labeled with FDAAs; cells were initially incubated 10 minutes with NADA, washed in PBS and resuspended in TSB. The cells were then incubated 10 minutes with HADA, washed with PBS, placed on an agarose pad and visualized by SR-SIM. This experiment was conducted in three biological replicates including a staining in reverse order and one using the red TADA as a replacement for NADA. Analysis on the progression of PG synthesis was performed on 300 cells for each biological replicate with similar results.

To investigate the progression of septal PG synthesis in *clpX* mutant cells displaying premature split, HADA incorporation was assessed in 50 cells (in each of three biological replicates) that had initiated septum formation during the initial labeling and displayed the characteristic morphology of premature splitting.

### FtsZ and PG synthesis localization by SR-SIM

In order to assess FtsZ relative to the active PG synthesis, *S*. *aureus* (8325–4) wild-type and *clpX* mutant transformed with pCQ11 expressing an eYFP-tagged derivative of FtsZ from an IPTG-inducible promoter were analyzed using sequentially labeling with FDAAs as described above (incubation with TADA for 10 minutes followed by HADA for 10 minutes). Cells were grown at 30°C in the presence of 50 μM IPTG (at higher IPTG concentrations cell division defects were observed in the wild-type strain).

### Genetic cross-over to delete *tarO* in SA564ΔclpX and JE2 ΔclpX

In order to assess if genetic inactivation of *tarO* rescues growth of *S*. *aureus clpX* mutants we attempted to delete the *tarO* from the chromosome as described in [56]. In brief, the pMAD pΔtagO plasmid was electroporated into SA564clpX and JE2clpX strains at 34°C instead of 30°C to reduce the risk of selecting for spontaneous suppressor mutants in the *clpX* strains [20]. To achieve integration into the chromosome by homologous recombination, cultures were grown at 42°C (non-permissive for plasmid replication) for 8 h before serially diluting and plating on TSA plates containing 5 mg ml^-1^ erythromycin. Plasmid integration into the chromosomal *tarO* locus was confirmed using primer sets P5: 5’—CTC CGT AAC AAA TTG AGG ATA ACA—3’and P4: 5’- TAG TCG TCC TCC TAA AAT ATA CTC– 3’ or P3: 5’—CCT AAG CCT GTT AAG TAA TCA TAT -3’ and P6: 5’—GAT CGA AGT TAG GCT GGT AAG A- 3’. A single colony was subsequently inoculated into 5 ml of TSB, and the culture was grown at the permissive temperature (34°C) in the absence of antibiotic selection to stimulate plasmid excision. At this stage, colony PCR, using primers P3: 5’—CCT AAG CCT GTT AAG TAA TCA TAT -3’ and P4 was performed to identify colonies that carried the chromosomal *tarO* deletion. In these cells, *tarO* is present on the pMAD plasmid and in order to lose the plasmid, cells were grown at the non-permissive temperature (37°C or 40°C as wild-type *tarO* mutants cannot grow at 42°C). We were, however, unable to achieve plasmid loss this way. Instead, we tried to select for *tarO* mutants by adding 1 μg ml^-1^ targocil (toxic to cells with an intact copy of *tarO*) to the growth medium, but all attempts to create *clpX*, *tarO* double mutants were unsuccessful [57].

### Statistical analysis

Statistical analysis was done using R statistical software. Student’s t-test was used to assess significant differences in growth in the absence or presence of a tested antibiotic. The Chi-squared test of independence was used to determine if there was a significant relationship between the proportion of cells assigned to each of the three phases or relevant phenotypes under the tested condition (number of cells in the relevant phase or phenotype/the total number of cells). A value P < 0.05 was considered significant.

## Supporting information

S1 Fig β-lactams stimulate growth of a *S*. *aureus clpX* deletion strains, but not of wild type or *clpP* deletion strains. Growth rates (h^-1^) for SA564, JE2, and Newman strains and corresponding *clpX* and *clpP* deletion mutants grown in the presence of increasing concentrations of β-lactams at 30°C. The average growth rate and standard deviations from three biological replicates were plotted; Numbers above bars indicate average doubling time in minutes. Asterisks indicate significantly improved growth (P < 0.05). The P values were obtained by comparing the growth rates at each antibiotic concentration to the growth rate without antibiotics and were calculated using Student’s t-test.
(TIF)

S2 Fig Muropeptide and PBP profiles in the *S*. *aureus* wild-type and *clpX* strain cells. **A)** Peptidoglycan structure in the SA564 wild type (black) and the SA564 *clpX* (grey) strains grown at 30˚C in the absence (upper panel) or presence of 0.02 ug ml^-1^ oxacillin (lower panel). The figure panel shows HPLC chromatograms of mutanolysin-digested peptidoglycan purified from the indicated strains and conditions, and peaks corresponding to monomers, dimers, trimers to higher oligomers have been assigned according to previous nomenclature [53] and as depicted below. **B)** PBP profiles in membranes derived from SA564 wild-type and SA564 *clpX* grown at 37˚ or 30˚C in the absence or presence of 0.05 μg ml^-1^ oxacillin, as indicated. PBPs were visualized by staining the purified membranes for 10 minutes with Bocillin-FL and separating proteins on a 7.5% SDS gel. The PBP4 levels in cells growing at 30˚C in the absence or presence of 0.05 ug ml^-1^ oxacillin (lower panel) were determined by Western blot analysis using PBP4 specific antibodies. The PBP4 Western was performed in three biological replicates with similar results.
(TIF)

S3 Fig Morphological changes in *S*. *aureus clpX* cells grown at 30°C. TEM and SEM images of *S*. *aureus* SA564 wild type cells (upper panel) and SA564 *clpX* cells (lower panel) harvested in exponential phase at 30°C. Note the many lysed cells in TEM images of the *clpX* mutant. Scale bar, 5.0 μm.
(TIF)

S4 Fig Oxacillin delays separation of daughter cells. TEM images of SA564 wild-type (panel **A** and **C**) or *clpX* cells (panel **B** and **D**) cells grown in TSB to mid-exponential phase at 30°C in the absence (panel **A** and **B**) or presence of 0.05 ug ml^-1^ oxacillin (panel **C** and **D**). The scale bar corresponds to 1.0 μm. The images show several features of β-lactam treated wild-type and *clpX* cells such as a weak or missing midline arrow (arrow A), a fuzzy cell wall appearance (arrow B), and cells failing to separate after division (arrow C). The asymmetrical septum ingrowth can still be observed in oxacillin treated the *clpX* mutant cells (arrow D).
(TIF)

S5 Fig PG synthesis in *S*. *aureus clpX* cells grown at 37°C follows the wild-type paradigm. SA564*clpX* cells were grown at 37°C in the absence of oxacillin and PG synthesis was followed by sequentially labeling with TADA (red, but displayed in magenta) for 10 min, followed by washing and labeling with HADA (blue, but displayed in cyan) for additional 10 min before cells were imaged using SR-SIM. The TADA and HADA signals do not overlap, illustrating that septal peptidoglycan synthesis is progressing predictably inwards and PG synthesis follows the wild-type paradigm for clpX cells in phase 1, 2 and 3 at 37°C. Images shown are representative of three biological replicates. Scale bar 1.0 μm.
(TIF)

S6 Fig FtsZ localization relative to PG synthesis in wild-type and clpX mutant. FtsZ localization was analyzed in *S*. *aureus* wild-type and *clpX* cells expressing an eYFP-tagged derivative of FtsZ expressed from an IPTG-inducible promoter. Localization of FtsZ relative to PG synthesis was analyzed by sequentially labeling *S*. *aureus* wild type and *clpX* cells growing in TSB supplemented with 50 uM IPTG at 30°C with TADA (displayed in magenta) for 10 minutes followed by washing and labeling with HADA (displayed in cyan) for additional 10 min prior to SR-SIM imaging. Images shown are representative of cells from three biological replicates. Scale bars, 1 μm (overview), 0.5 μm (single cells).
(TIF)

S7 Fig Effect of different antibiotics on growth of the wild type and *clpX* cells. *S*. *aureus* SA564 wild type and *clpX* strains were grown overnight at 37°C, diluted 1:200 and grown at 37°C until mid-exponential phase. These cultures were then diluted into TSB containing increasing concentrations of the indicated compounds in a 96-well format, and the plates were incubated for 24 h at 30°C. The values represent means of OD values, normalized to the OD values obtained without compound. Error bars indicate standard deviations. Note that different scales were used on the two axes due to the difference in growth between the WT and *clpX* mutant: values for the *clpX* mutant are indicated on the left vertical axis, and values for the WT are indicated on the right vertical axis to allow easy comparison of growth between the two strains. (**A)** *S*. *aureus* wild type and *clpX* mutant grown in the presence of various antibiotics and PG synthesis inhibitors.(**B**) *S*. *aureus* wild type and *clpX* mutant grown in the presence of β-lactams with different PBP specificity: meropenem, imipenem, and cloxacillin are specific for PBP1; cephradine binds preferentially to PBP1 and PBP3; cefuroxime an cefotaxime bind preferentially to PBP2, and cefoxitin is specific for PBP4 [37–40]. Oxacillin is non-specific and targets multiple PBPs.
(TIF)

S1 Movie Oxacillin rescues growth of the *S*. *aureus clpX* mutant. Time lapse microscopy of SA564 and SA564ΔclpX growing at 30°C in the absence or presence of 0.01 μg ml^-1^ oxacillin. In the middle panel (T = -90) cells were exposed to oxacillin 90 min prior to imaging; in the lower panel (T = 0) *clpX* cells were exposed to oxacillin, when transferred to the agarose pad used for imaging. Before imaging, cells were first inoculated in TSB and grown at 37°C for four generations before shifting cells to 30°C and growing for 90 min in the absence or presence of oxacillin. Scale bar, 5 μm.
(AVI)

S2 Movie FtsZ localization and Z-ring dynamics appear similar in *S*. *aureus* wild-type and *clpX* mutant cells. Time lapse fluorescence microscopy was used to study FtsZ localization and dynamics *S*. *aureus* (8325–4) wild-type and *clpX* mutant expressing an eYFP-tagged derivative of FtsZ from an IPTG-inducible promoter and growing on a semi-solid matrix in the presence of 100 uM IPTG at 30°C. The fluorescent signal is overlaid with the phase contrast image, scale bar 1 μm.
(AVI)

S1 Table Bacterial strains used in this study. (DOCX)

S2 Table Experimental specifications used in SR-SIM. (DOCX)

S3 Table Fluorescent dyes used in SR-SIM. (DOCX)
