# Supplementary figures and images for: The ClpX chaperone controls autolytic splitting of Staphylococcus aureus daughter cells, but is bypassed by β-lactam antibiotics or inhibitors of WTA biosynthesis
Source: PLoS Pathog. 2019 Sep 13;15(9):e1008044. doi: 10.1371/journal.ppat.1008044 (PMC6760813; doi:10.1371/journal.ppat.1008044)

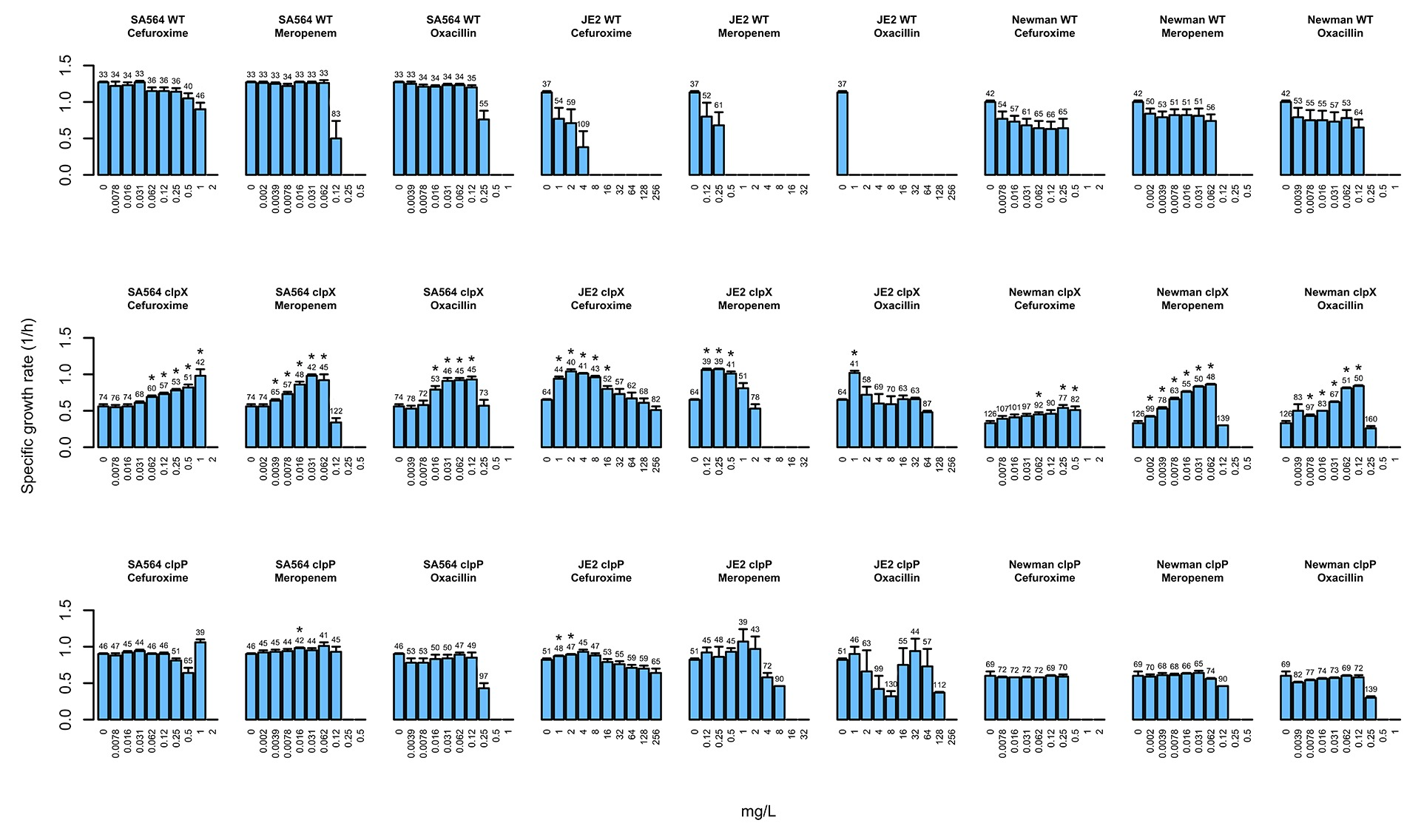

Supplement: S1 Fig — Growth rates (h-1) for SA564, JE2, and Newman strains and corresponding clpX and clpP deletion mutants grown in the presence of increasing concentrations of β-lactams at 30°C. The average growth rate and standard deviations from three biological replicates were plotted; Numbers above bars indicate average doubling time in minutes. Asterisks indicate significantly improved growth (P < 0.05). The P values were obtained by comparing the growth rates at each antibiotic concentration to the growth rate without antibiotics and were calculated using Student’s t-test. (TIF) [file ppat.1008044.s001.tif]

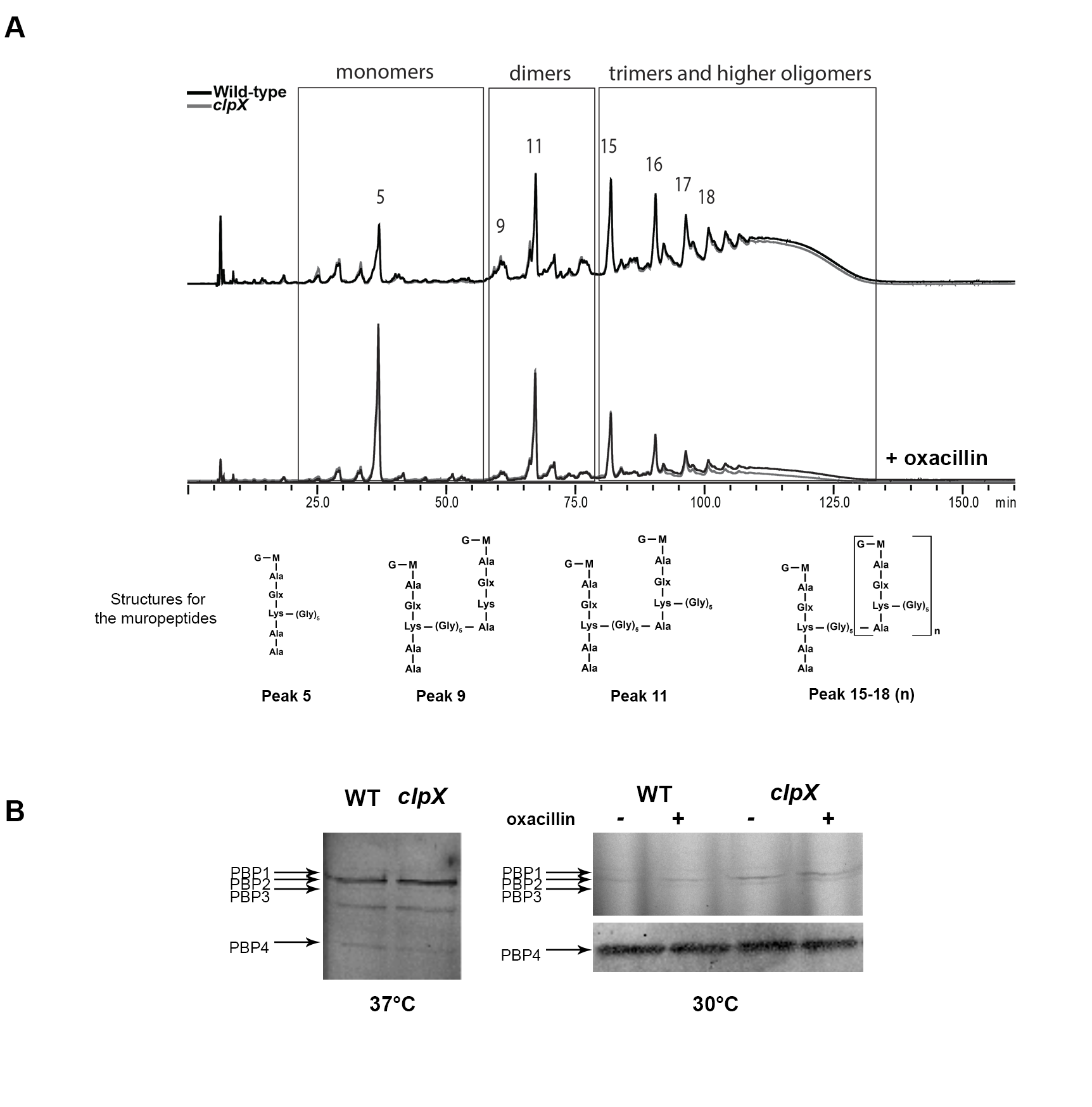

Supplement: S2 Fig — A) Peptidoglycan structure in the SA564 wild type (black) and the SA564 clpX (grey) strains grown at 30˚C in the absence (upper panel) or presence of 0.02 ug ml-1 oxacillin (lower panel). The figure panel shows HPLC chromatograms of mutanolysin-digested peptidoglycan purified from the indicated strains and conditions, and peaks corresponding to monomers, dimers, trimers to higher oligomers have been assigned according to previous nomenclature [53] and as depicted below. B) PBP profiles in membranes derived from SA564 wild-type and SA564 clpX grown at 37˚ or 30˚C in the absence or presence of 0.05 μg ml-1 oxacillin, as indicated. PBPs were visualized by staining the purified membranes for 10 minutes with Bocillin-FL and separating proteins on a 7.5% SDS gel. The PBP4 levels in cells growing at 30˚C in the absence or presence of 0.05 ug ml-1 oxacillin (lower panel) were determined by Western blot analysis using PBP4 specific antibodies. The PBP4 Western was performed in three biological replicates with similar results. (TIF) [file ppat.1008044.s002.tif]

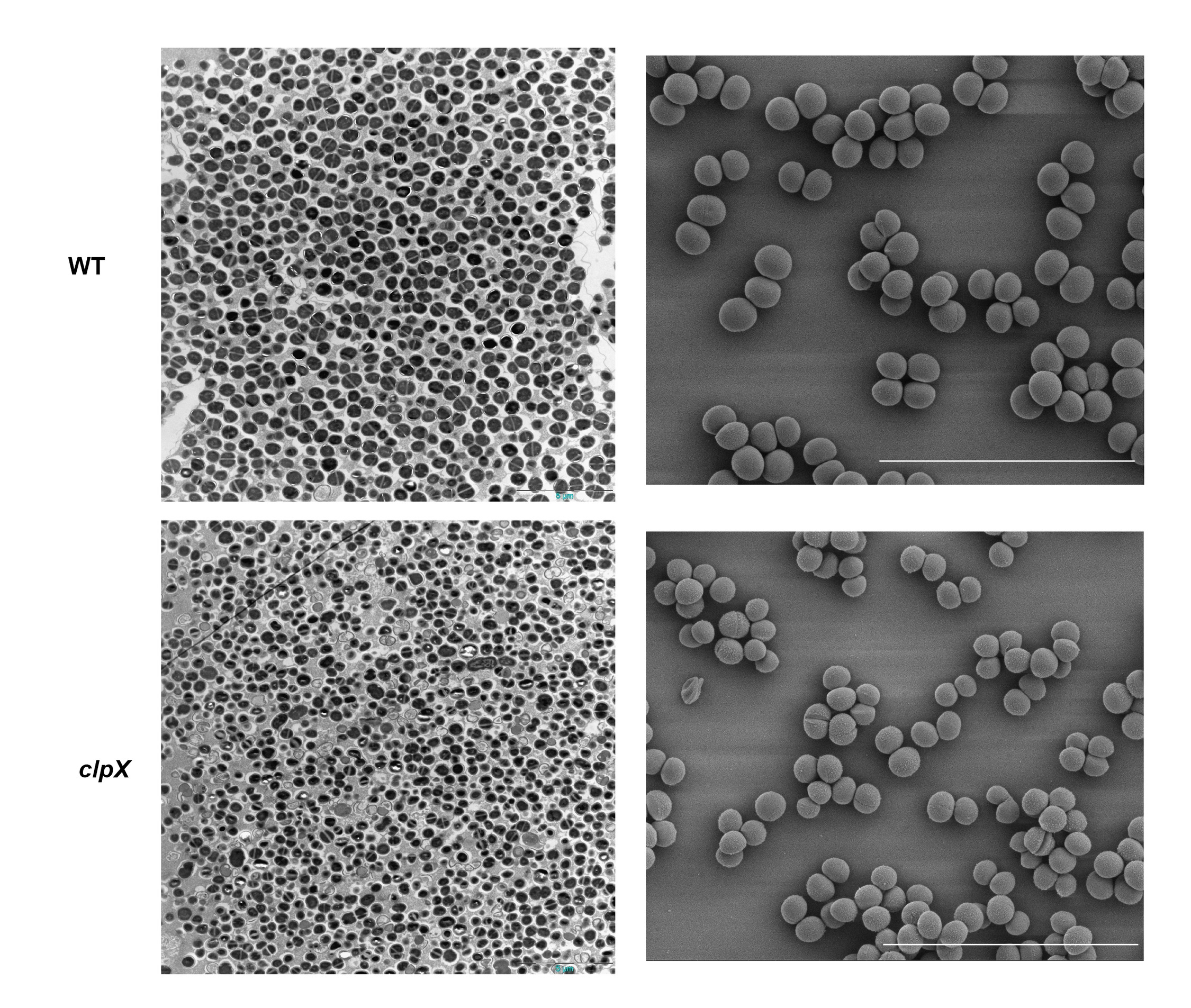

Supplement: S3 Fig — TEM and SEM images of S. aureus SA564 wild type cells (upper panel) and SA564 clpX cells (lower panel) harvested in exponential phase at 30°C. Note the many lysed cells in TEM images of the clpX mutant. Scale bar, 5.0 μm. (TIF) [file ppat.1008044.s003.tif]

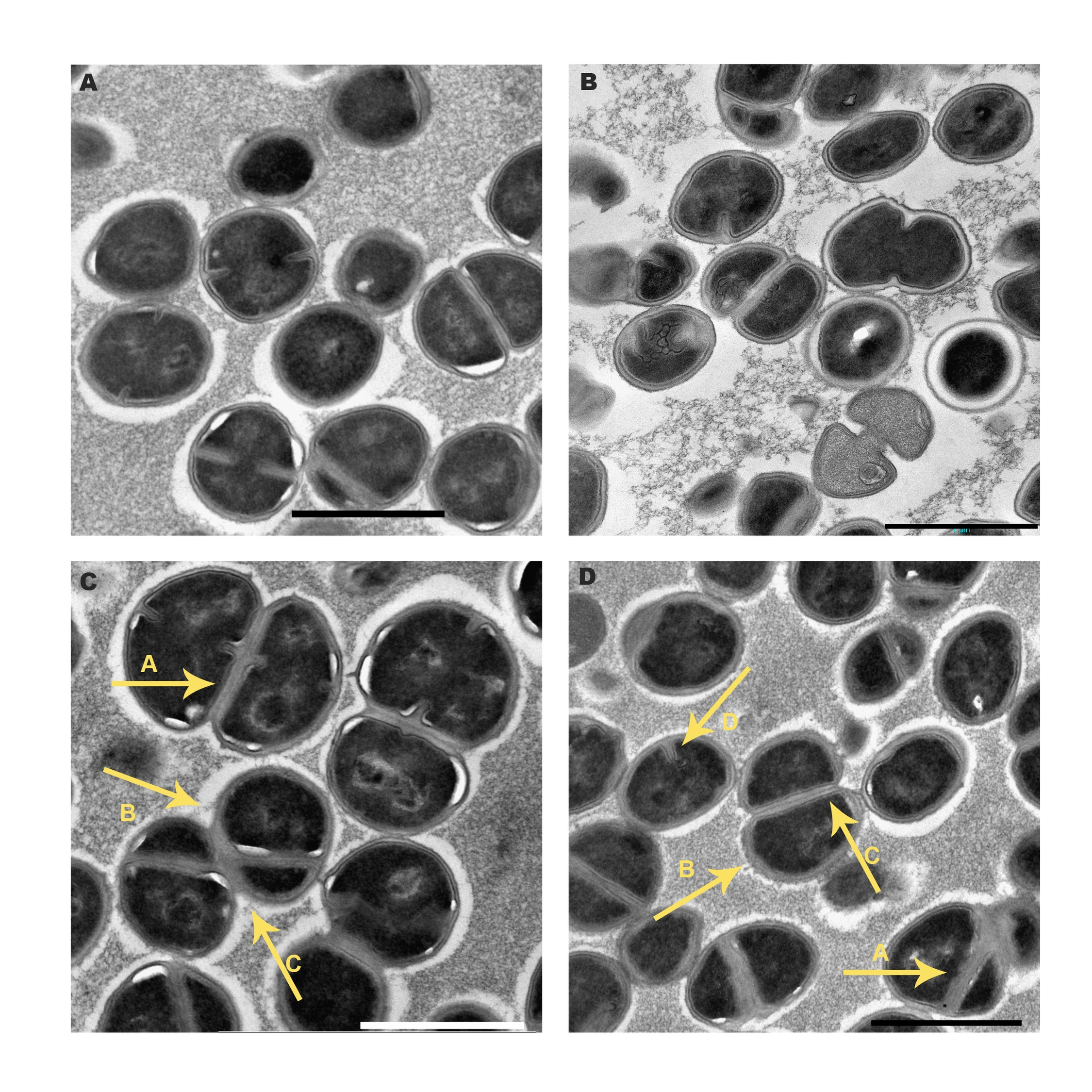

Supplement: S4 Fig — TEM images of SA564 wild-type (panel A and C) or clpX cells (panel B and D) cells grown in TSB to mid-exponential phase at 30°C in the absence (panel A and B) or presence of 0.05 ug ml-1 oxacillin (panel C and D). The scale bar corresponds to 1.0 μm. The images show several features of β-lactam treated wild-type and clpX cells such as a weak or missing midline arrow (arrow A), a fuzzy cell wall appearance (arrow B), and cells failing to separate after division (arrow C). The asymmetrical septum ingrowth can still be observed in oxacillin treated the clpX mutant cells (arrow D). (TIF) [file ppat.1008044.s004.tif]

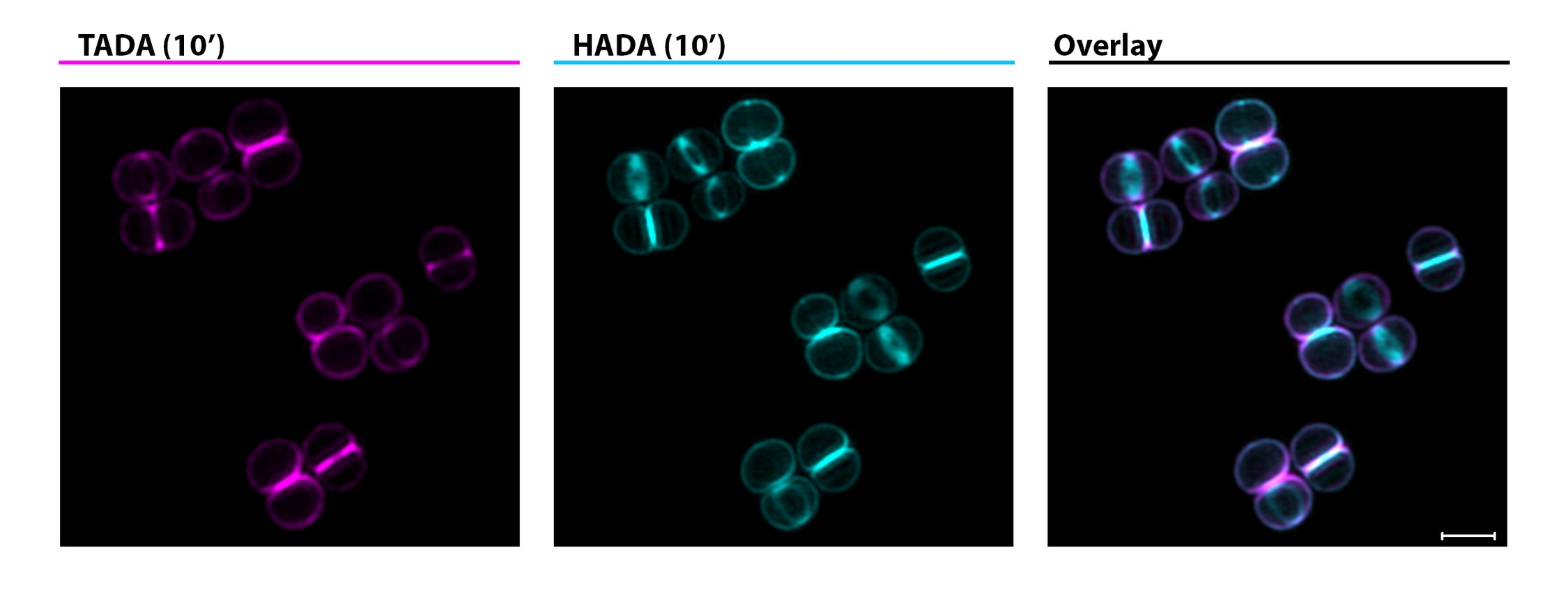

Supplement: S5 Fig — SA564clpX cells were grown at 37°C in the absence of oxacillin and PG synthesis was followed by sequentially labeling with TADA (red, but displayed in magenta) for 10 min, followed by washing and labeling with HADA (blue, but displayed in cyan) for additional 10 min before cells were imaged using SR-SIM. The TADA and HADA signals do not overlap, illustrating that septal peptidoglycan synthesis is progressing predictably inwards and PG synthesis follows the wild-type paradigm for clpX cells in phase 1, 2 and 3 at 37°C. Images shown are representative of three biological replicates. Scale bar 1.0 μm. (TIF) [file ppat.1008044.s005.tif]

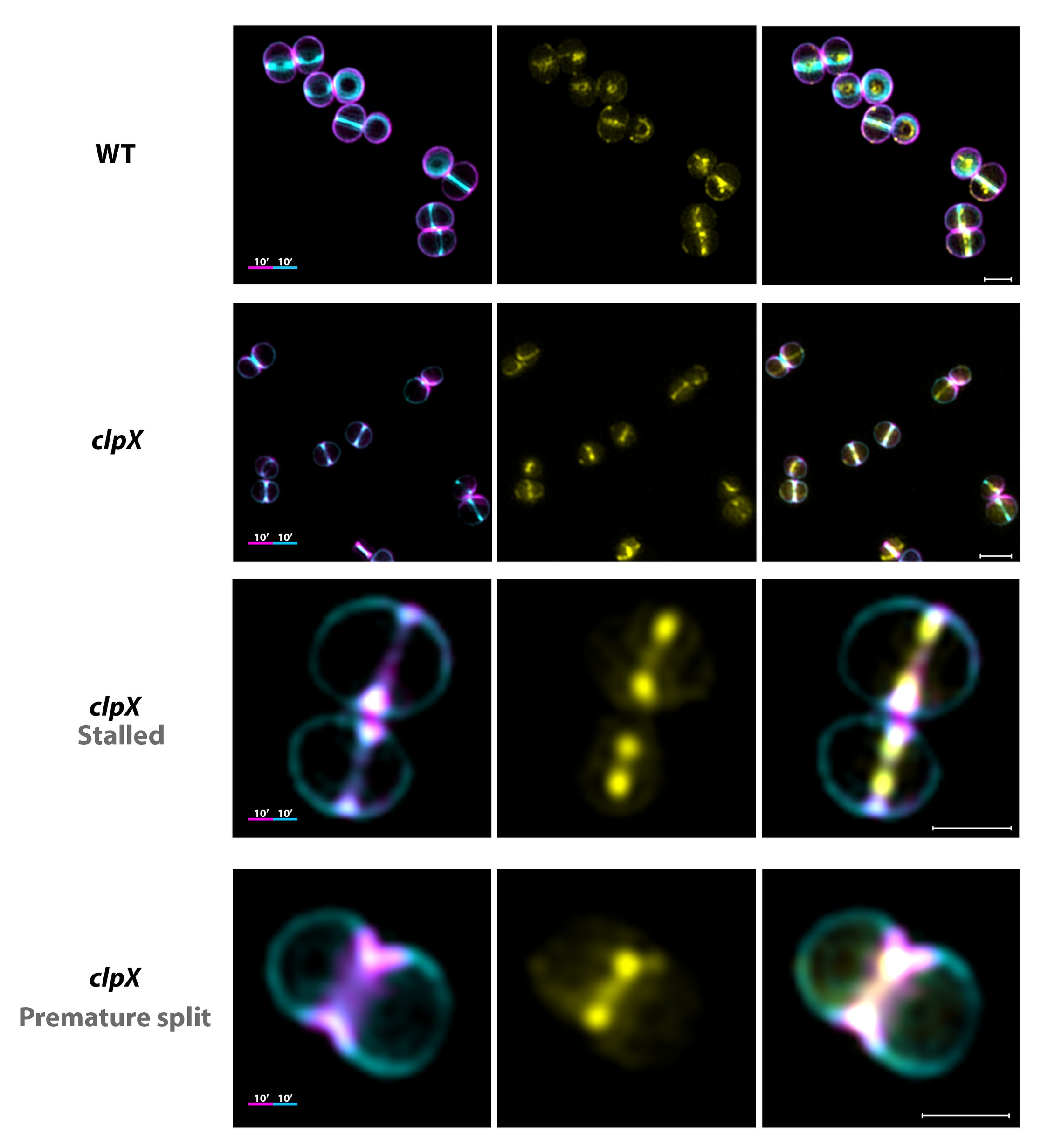

Supplement: S6 Fig — FtsZ localization was analyzed in S. aureus wild-type and clpX cells expressing an eYFP-tagged derivative of FtsZ expressed from an IPTG-inducible promoter. Localization of FtsZ relative to PG synthesis was analyzed by sequentially labeling S. aureus wild type and clpX cells growing in TSB supplemented with 50 uM IPTG at 30°C with TADA (displayed in magenta) for 10 minutes followed by washing and labeling with HADA (displayed in cyan) for additional 10 min prior to SR-SIM imaging. Images shown are representative of cells from three biological replicates. Scale bars, 1 μm (overview), 0.5 μm (single cells). (TIF) [file ppat.1008044.s006.tif]

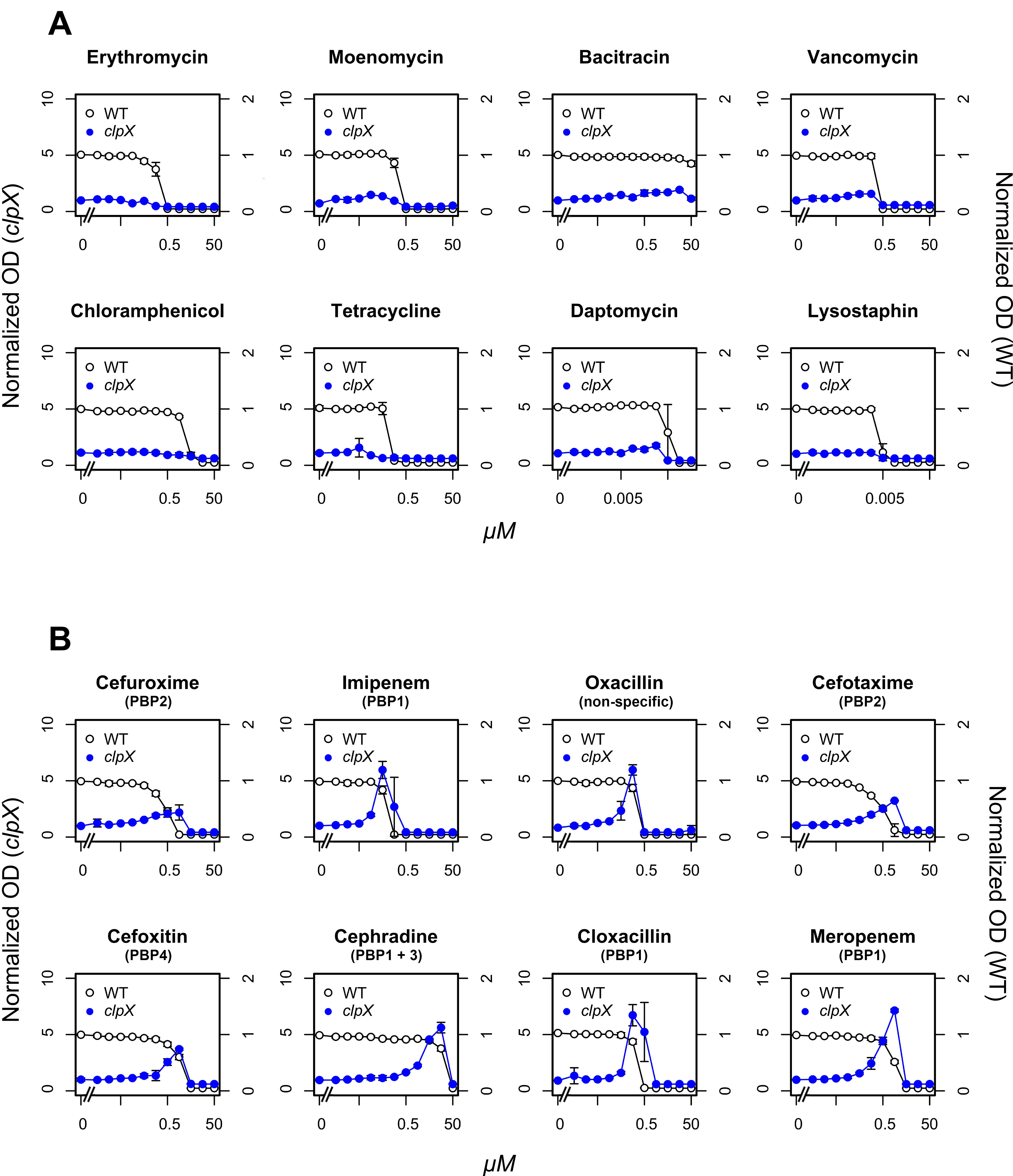

Supplement: S7 Fig — S. aureus SA564 wild type and clpX strains were grown overnight at 37°C, diluted 1:200 and grown at 37°C until mid-exponential phase. These cultures were then diluted into TSB containing increasing concentrations of the indicated compounds in a 96-well format, and the plates were incubated for 24 h at 30°C. The values represent means of OD values, normalized to the OD values obtained without compound. Error bars indicate standard deviations. Note that different scales were used on the two axes due to the difference in growth between the WT and clpX mutant: values for the clpX mutant are indicated on the left vertical axis, and values for the WT are indicated on the right vertical axis to allow easy comparison of growth between the two strains. (A) S. aureus wild type and clpX mutant grown in the presence of various antibiotics and PG synthesis inhibitors.(B) S. aureus wild type and clpX mutant grown in the presence of β-lactams with different PBP specificity: meropenem, imipenem, and cloxacillin are specific for PBP1; cephradine binds preferentially to PBP1 and PBP3; cefuroxime an cefotaxime bind preferentially to PBP2, and cefoxitin is specific for PBP4 [37–40]. Oxacillin is non-specific and targets multiple PBPs. (TIF) [file ppat.1008044.s007.tif]
