# Supplementary material for: The ClpX chaperone controls autolytic splitting of Staphylococcus aureus daughter cells, but is bypassed by β-lactam antibiotics or inhibitors of WTA biosynthesis
Source: PLoS Pathog. 2019 Sep 13;15(9):e1008044. doi: 10.1371/journal.ppat.1008044 (PMC6760813; doi:10.1371/journal.ppat.1008044)
Supplement: S1 Table — (DOCX) [file ppat.1008044.s010.docx]

**S1 Table: Bacterial strains used in this study**

| Strain | Description | Source |
| --- | --- | --- |
| SA564 | MSSA; low-passage clinical isolate | [1] |
| SA564Δ*clpX* | 651 bp in-frame deletion in the *clpX* gene | [2] |
| SA564Δ*clpP* | The entire *clpP* gene was deleted | [3] |
| SA564Δ*clpX atl* | The *atl*::tn was transduced from ANG406 into SA564Δ*clpX* using phage 85 | This study |
| SA564Δ*clpX sle1* | The *sle1:*:tn was transduced from the NARSA-library (SAUSA300_0438) into SA564Δ*clpX* using phage 85 | This study |
| SA564*atl* | The *atl*::tn was transduced from ANG406 into SA564 wild-type using phage 85 | This study |
| SA564*sle1* | The *sle1*::tn was transduced from the NARSA-library (SAUSA300_0438) into SA564 wild-type using phage 85 | This study |
| JE2 | CA-MRSA strain USA300 LAC cured of plasmids | [4] |
| JE2Δ*clpX* | 651 bp in-frame deletion in the *clpX* gene | [5] |
| JE2*clpP* | *bursa aurealis* transposon mutant, Em^R^ | [5] |
| JE2*sle1* | SAUSA300_0438; *bursa aurealis* transposon mutant | [4] |
| 8325-4 | MSSA strain cured of prophages | [7] |
| 8325-4Δ*clpX* | 651 bp in-frame deletion in the *clpX* gene | [9] |
| 8325-4Δ*clpP* | The entire *clpP* gene was deleted | [8] |
| Newman | MSSA, preferred lab strain of clinical origin(ATCC 25904) | [9] |
| NewmanΔ*clpX* | 651 bp in-frame deletion in the clpX gene | [10] |
| NewmanΔ*clpP* | The entire clpP gene was deleted | [3] |
| ANG506 | SEJ1Δ*atl*; *bursa aurealis* transposon mutant, Em^R^ | [6] |

**References**

1. Somerville, G. A. *et al*. In vitro serial passage of *Staphylococcus aureus*: changes in physiology, virulence factor production, and *agr* nucleotide sequence. J Bacteriol. 2002;184: 1430–1437.

2. Jelsbak, L. *et al*. The chaperone ClpX stimulates expression of *Staphylococcus aureus* protein A by Rot dependent and independent pathways. PLoS ONE 2010; 5: e12752.

3. Frees, D. *et al*. New insights into *Staphylococcus aureus* stress tolerance and virulence regulation from an analysis of the role of the ClpP protease in the strains Newman, COL, and SA564. J Proteome Res. 2012;11: 95–108.

4. 8. Fey, P. D. *et al*. A genetic resource for rapid and comprehensive phenotype screening of nonessential *Staphylococcus aureus* genes. MBio 2013; **4**: e00537–12.

5. Bæk, K. T$.$ *et al*. β-lactam resistance in methicillin-resistant *Staphylococcus aureus* USA300 is increased by inactivation of the ClpXP protease. Antimicrob Agents Chemother. 2014;58: 4593–4603.

6. Corrigan RM, Abbott JC, Burhenne H, Kaever V, Gründling A.. c-di-AMP is a new second messenger in Staphylococcus aureus with a role in controlling cell size and envelope stress. PLoS Pathog. 2011;7: e1002217.

7. Bæk, K. T. *et al*. Genetic variation in the *Staphylococcus aureus* 8325 strain lineage revealed by whole-genome sequencing. PLoS ONE 2013; 8: e77122.

8. Frees, D., Qazi, S., Hill, P. J. & Ingmer, H. Alternative roles of ClpX and ClpP in *Staphylococcus aureus* stress tolerance and virulence. Mol Microbiol. 2002;48: 1565–1578.

9. Duthie, E. S. & Lorenz, L. L. Staphylococcal coagulase; mode of action and antigenicity. J Gen Microbiol. 1952;6: 95–107.

10. Bæk, K. T. *et al*. The cell wall polymer lipoteichoic acid becomes non-essential in *Staphylococcus aureus* cells lacking the ClpX chaperone. MBio 2016;7: e01228
