# Supplementary material for: The ClpX chaperone controls autolytic splitting of Staphylococcus aureus daughter cells, but is bypassed by β-lactam antibiotics or inhibitors of WTA biosynthesis
Source: PLoS Pathog. 2019 Sep 13;15(9):e1008044. doi: 10.1371/journal.ppat.1008044 (PMC6760813; doi:10.1371/journal.ppat.1008044)
Supplement: S2 Table — (DOCX) [file ppat.1008044.s011.docx]

**S2 Table: Experimental specifications used in SR-SIM.**

| Laser | Type & Power | Beam splitter | Grating | Dyes |
| --- | --- | --- | --- | --- |
| 405 nm | HR Diode – 50mW | BP 420-480 + LP 750 | 23 µm | HADA & Hoechst |
| 488 nm | HR Diode – 50mW | BP 495-575 + LP 750 | 28 µm | WGA, Van-fl & NADA |
| 561 nm | HR Diode – 100mW | BP 570-650 + LP 750 | 34 µm | Nile Red, TADA |
