# Supplementary material for: The ClpX chaperone controls autolytic splitting of Staphylococcus aureus daughter cells, but is bypassed by β-lactam antibiotics or inhibitors of WTA biosynthesis
Source: PLoS Pathog. 2019 Sep 13;15(9):e1008044. doi: 10.1371/journal.ppat.1008044 (PMC6760813; doi:10.1371/journal.ppat.1008044)
Supplement: S3 Table — (DOCX) [file ppat.1008044.s012.docx]

| Dye | Target | Concentration | Laser | Laser power | Exposue |
| --- | --- | --- | --- | --- | --- |
| Nile Red | Membrane | 5 µg/ml | 561 nm | 5 % | 50 ms |
| WGA-488 | Cell wall (lateral) | 1 µg/ml | 488 nm | 20 % | 50 ms |
| Van-fl* | Cell wall | 0.8 µg/ml | 488 nm | 20 % | 100 ms |
| Hoechst | DNA (all cells) | 1 µg/ml | 405 nm | 50 % | 100 ms |
| NADA | Active PG synthesis | 250 µM | 488 nm | 20 % | 50 ms |
| TADA | Active PG synthesis | 250 µM | 561 nm | 20 % | 100 ms |
| HADA | Active PG synthesis | 250 µM | 405 nm | 50 % | 300 ms |

**S3 Table: Fluorescent dyes used in SR-SIM.**

* Van-FL solution comprises a mixture containing equal amounts of vancomycin (Sigma) and a BODIPY FL conjugate of vancomycin to a final concentration of 0.8 μg ml^-1^.
